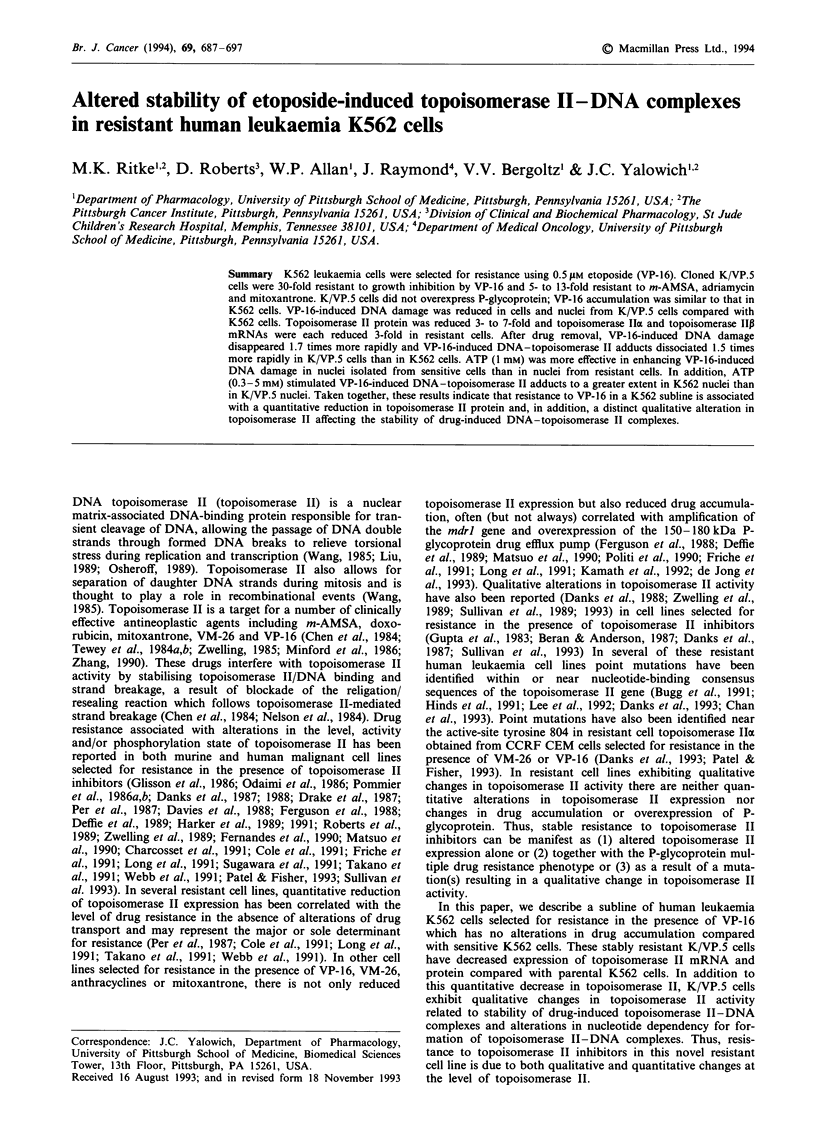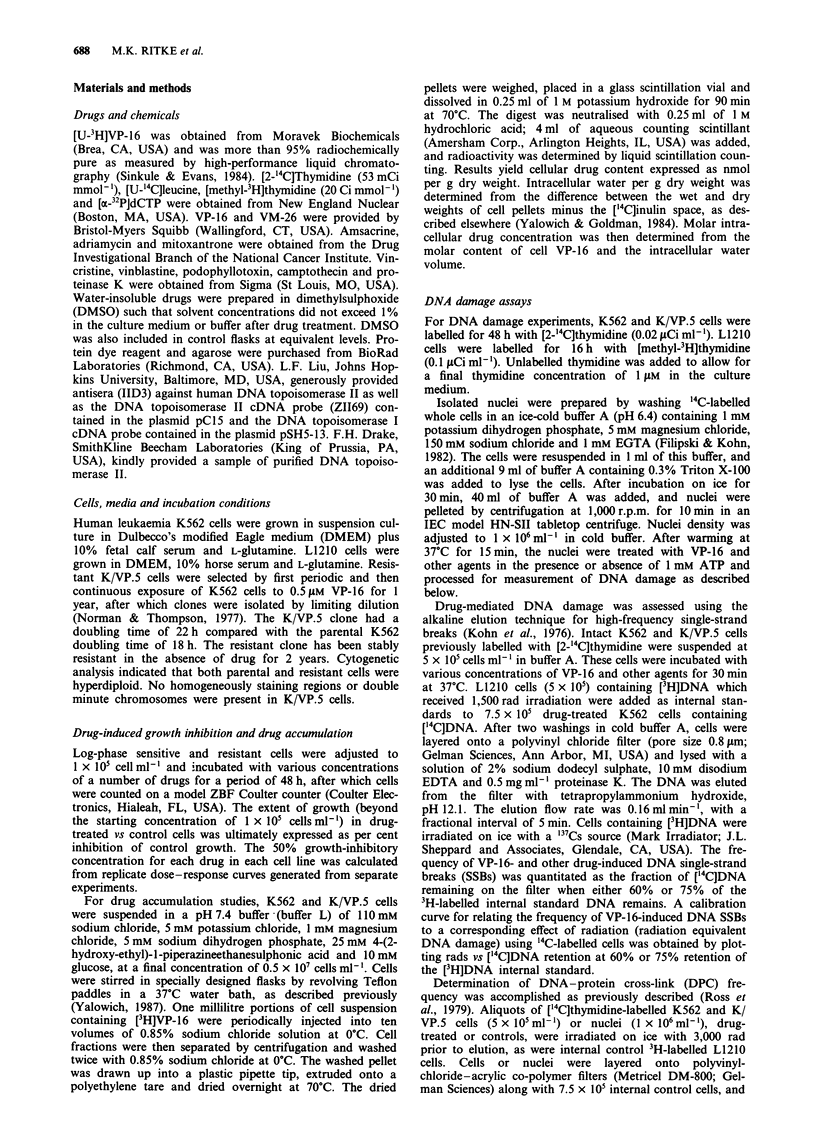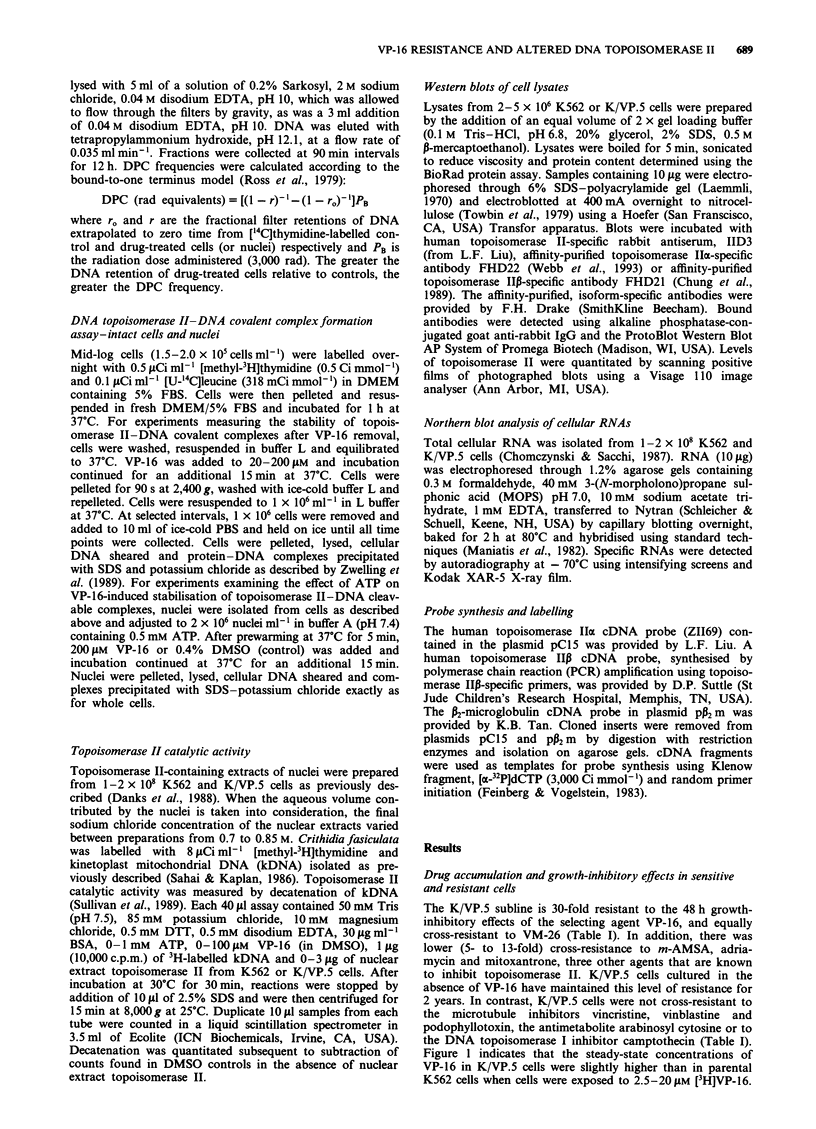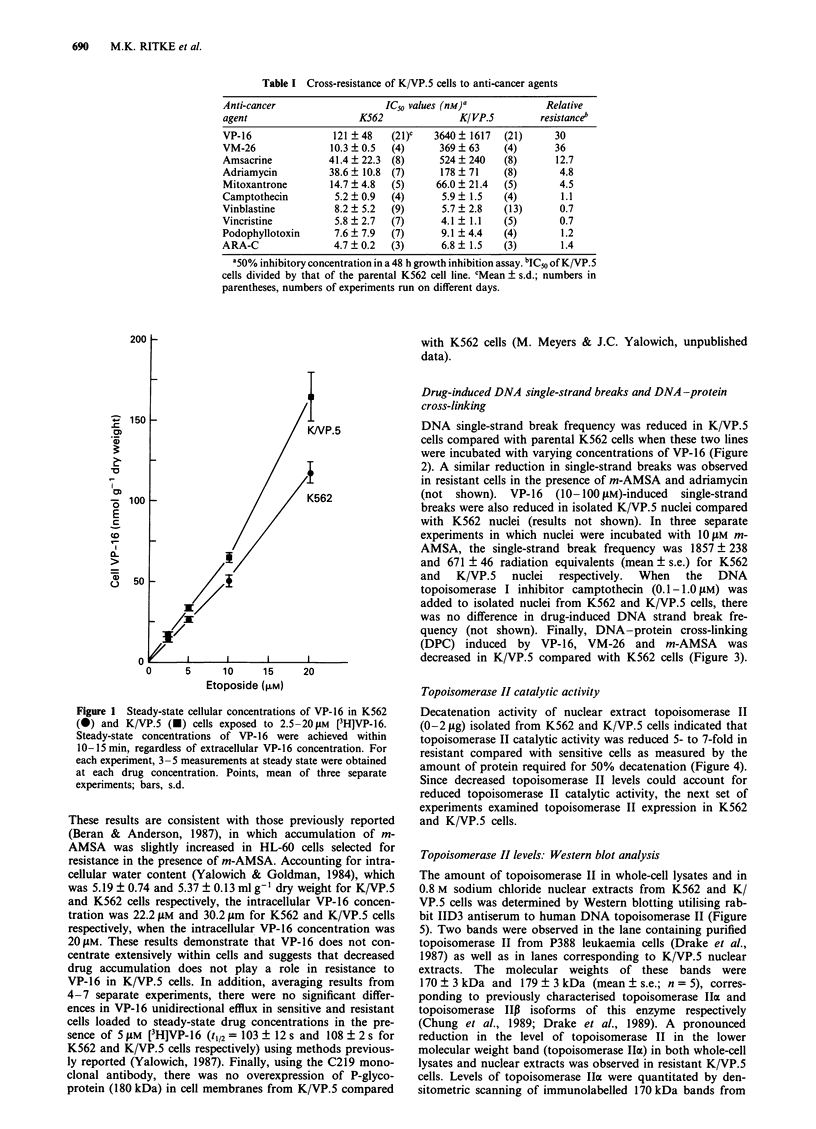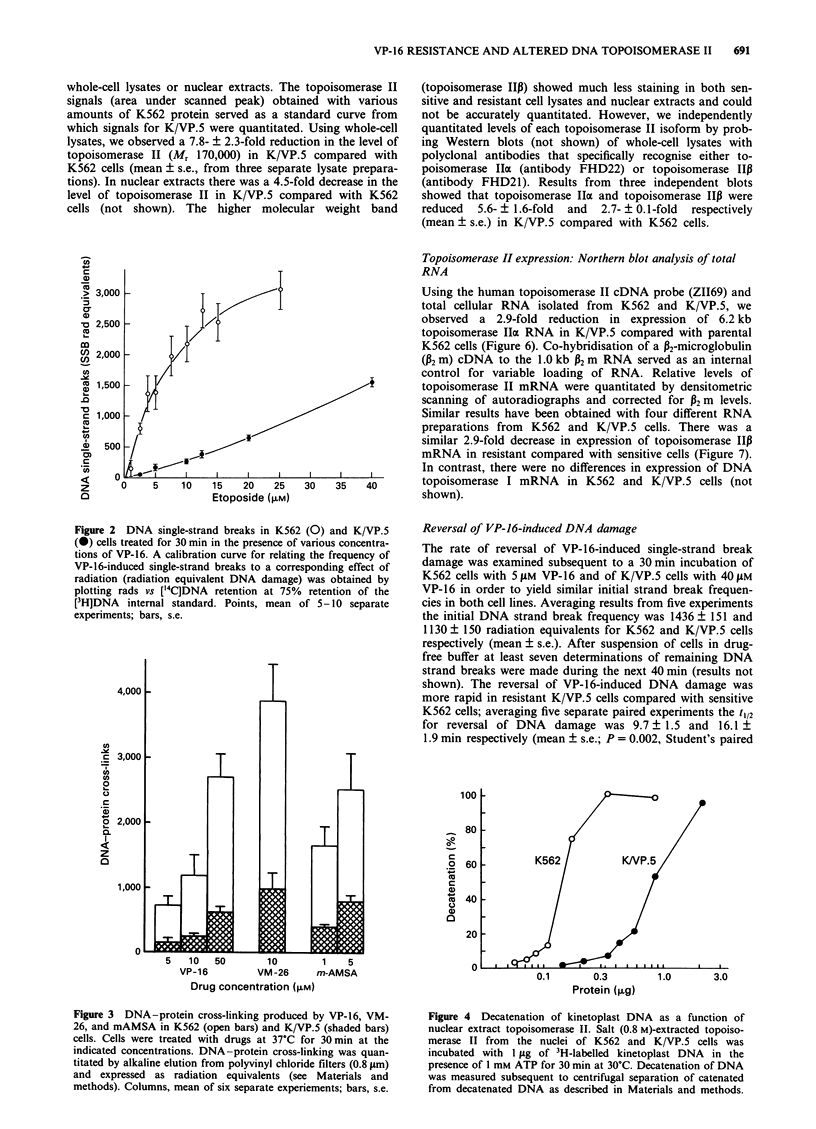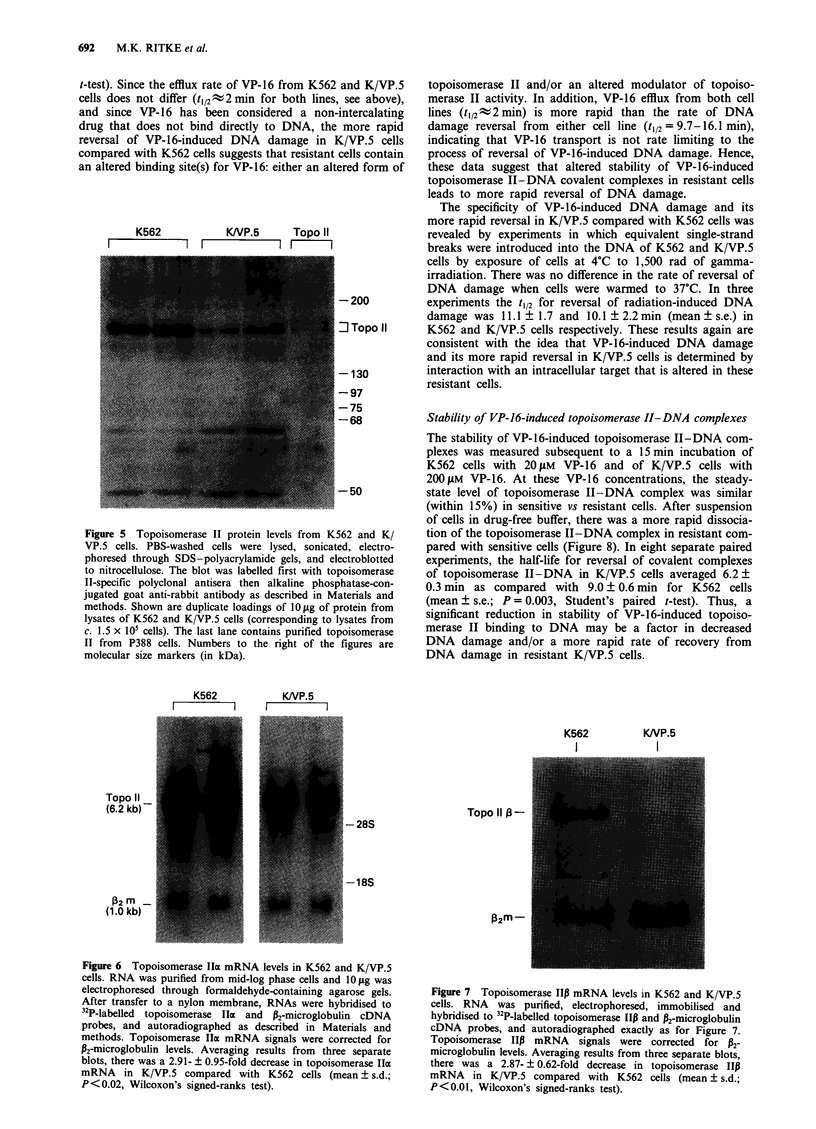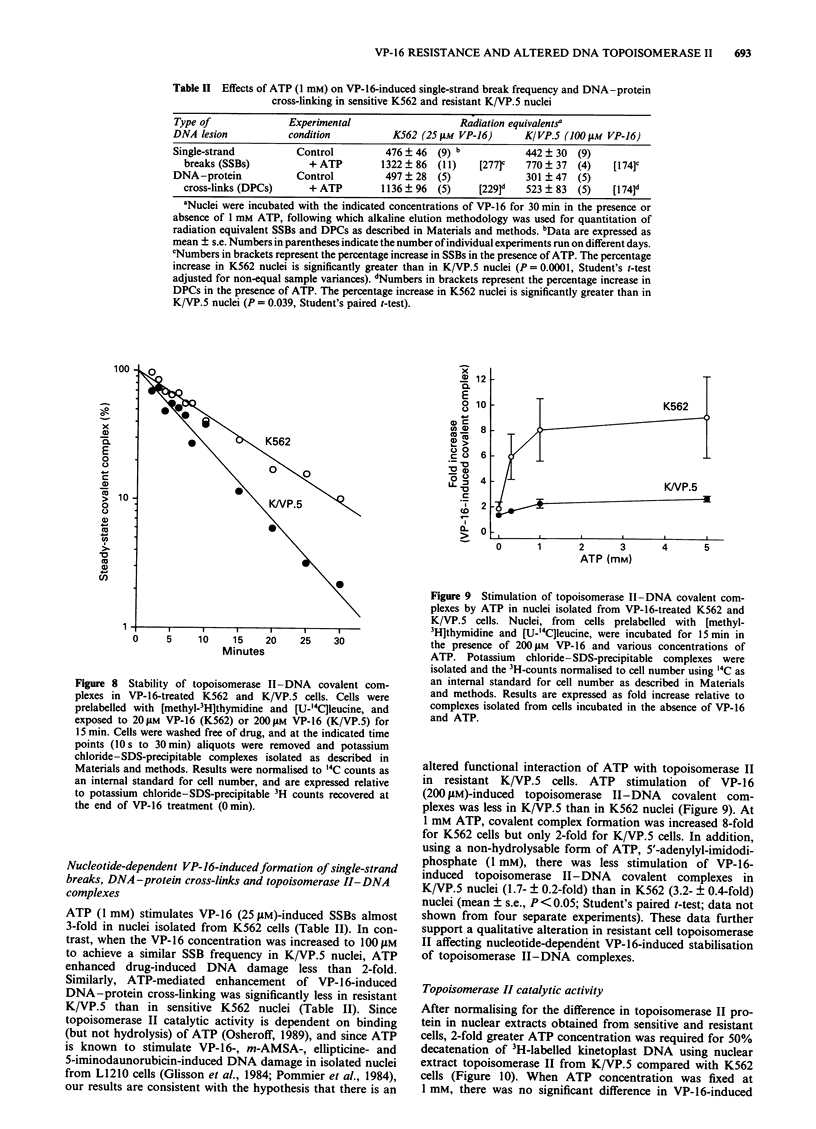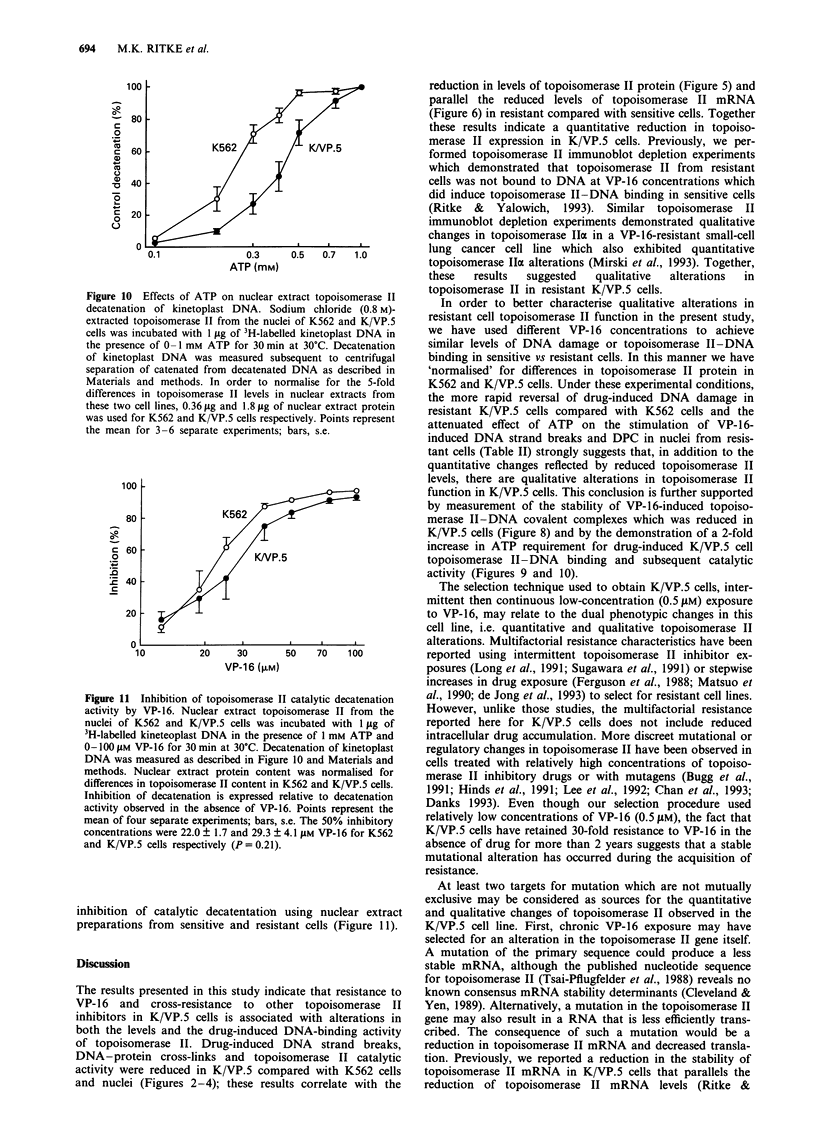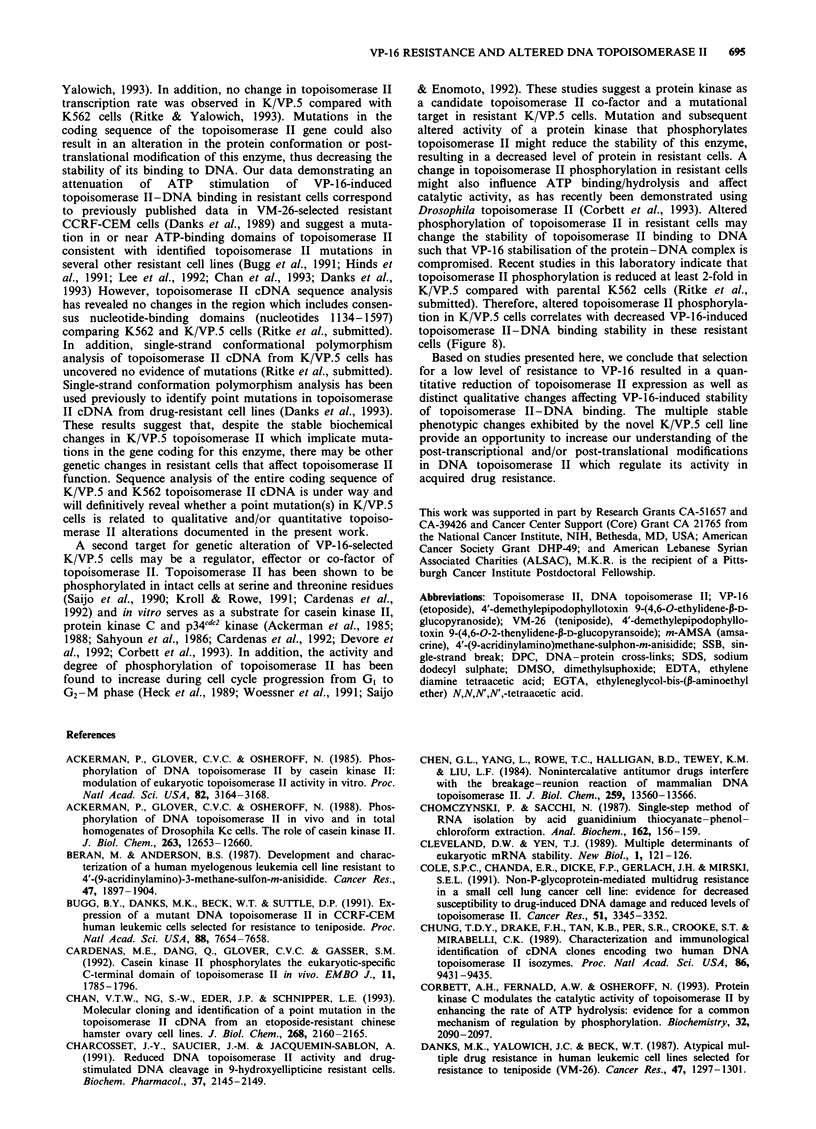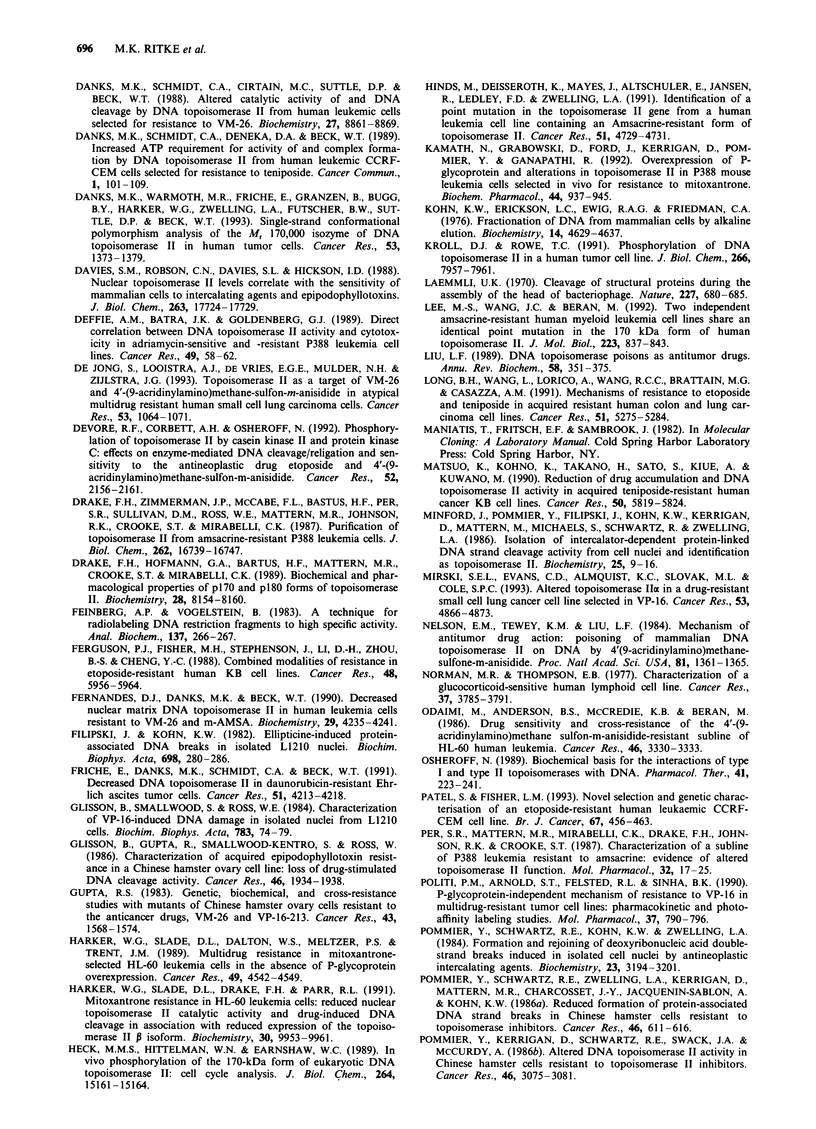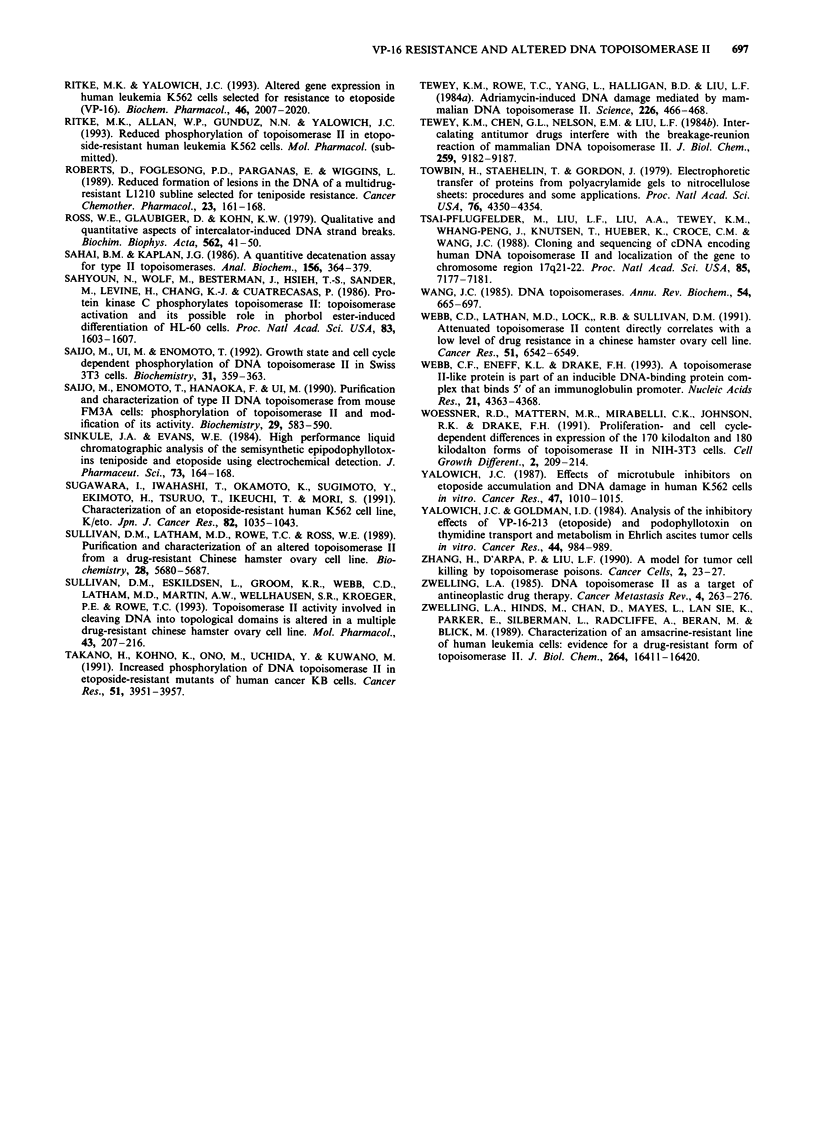# Altered stability of etoposide-induced topoisomerase II-DNA complexes in resistant human leukaemia K562 cells.

**DOI:** 10.1038/bjc.1994.131

**Published:** 1994-04

**Authors:** M. K. Ritke, D. Roberts, W. P. Allan, J. Raymond, V. V. Bergoltz, J. C. Yalowich

**Affiliations:** Department of Pharmacology, University of Pittsburgh School of Medicine, Pennsylvania 15261.

## Abstract

**Images:**


Br. J. Cancer (1994), 69, 687-697 © Macmillan Press Ltd., 1994

### Altered stability of etoposide-induced topoisomerase II-DNA complexes in resistant human leukaemia K562 cells

M.K. Ritke[1,2], D. Roberts[3], W.P. Allan[1], J. Raymond[4], V.V. Bergoltz[1] & J.C. Yalowich[1,2]

[1]*Department of Pharmacology, University of Pittsburgh School of Medicine, Pittsburgh, Pennsylvania 15261, USA;* [2]*The Pittsburgh Cancer Institute, Pittsburgh, Pennsylvania 15261, USA;* [3]*Division of Clinical and Biochemical Pharmacology, St Jude Children's Research Hospital, Memphis, Tennessee 38101, USA;* [4]*Department of Medical Oncology, University of Pittsburgh School of Medicine, Pittsburgh, Pennsylvania 15261, USA.*

**Summary** K562 leukaemia cells were selected for resistance using 0.5 μM etoposide (VP-16). Cloned K/VP.5 cells were 30-fold resistant to growth inhibition by VP-16 and 5- to 13-fold resistant to *m*-AMSA, adriamycin and mitoxantrone. K/VP.5 cells did not overexpress P-glycoprotein; VP-16 accumulation was similar to that in K562 cells. VP-16-induced DNA damage was reduced in cells and nuclei from K/VP.5 cells compared with K562 cells. Topoisomerase II protein was reduced 3- to 7-fold and topoisomerase IIα and topoisomerase IIβ mRNAs were each reduced 3-fold in resistant cells. After drug removal, VP-16-induced DNA damage disappeared 1.7 times more rapidly and VP-16-induced DNA-topoisomerase II adducts dissociated 1.5 times more rapidly in K/VP.5 cells than in K562 cells. ATP (1 mM) was more effective in enhancing VP-16-induced DNA damage in nuclei isolated from sensitive cells than in nuclei from resistant cells. In addition, ATP (0.3-5 mM) stimulated VP-16-induced DNA-topoisomerase II adducts to a greater extent in K562 nuclei than in K/VP.5 nuclei. Taken together, these results indicate that resistance to VP-16 in a K562 subline is associated with a quantitative reduction in topoisomerase II protein and, in addition, a distinct qualitative alteration in topoisomerase II affecting the stability of drug-induced DNA-topoisomerase II complexes.

DNA topoisomerase II (topoisomerase II) is a nuclear matrix-associated DNA-binding protein responsible for transient cleavage of DNA, allowing the passage of DNA double strands through formed DNA breaks to relieve torsional stress during replication and transcription (Wang, 1985; Liu, 1989; Osheroff, 1989). Topoisomerase II also allows for separation of daughter DNA strands during mitosis and is thought to play a role in recombinational events (Wang, 1985). Topoisomerase II is a target for a number of clinically effective antineoplastic agents including *m*-AMSA, doxorubicin, mitoxantrone, VM-26 and VP-16 (Chen *et al.*, 1984; Tewey *et al.*, 1984*a,b*; Zwelling, 1985; Minford *et al.*, 1986; Zhang, 1990). These drugs interfere with topoisomerase II activity by stabilising topoisomerase II/DNA binding and strand breakage, a result of blockade of the religation/resealing reaction which follows topoisomerase II-mediated strand breakage (Chen *et al.*, 1984; Nelson *et al.*, 1984). Drug resistance associated with alterations in the level, activity and/or phosphorylation state of topoisomerase II has been reported in both murine and human malignant cell lines selected for resistance in the presence of topoisomerase II inhibitors (Glisson *et al.*, 1986; Odaimi *et al.*, 1986; Pommier *et al.*, 1986*a,b*; Danks *et al.*, 1987; 1988; Drake *et al.*, 1987; Per *et al.*, 1987; Davies *et al.*, 1988; Ferguson *et al.*, 1988; Deffie *et al.*, 1989; Harker *et al.*, 1989; 1991; Roberts *et al.*, 1989; Zwelling *et al.*, 1989; Fernandes *et al.*, 1990; Matsuo *et al.*, 1990; Charcosset *et al.*, 1991; Cole *et al.*, 1991; Friche *et al.*, 1991; Long *et al.*, 1991; Sugawara *et al.*, 1991; Takano *et al.*, 1991; Webb *et al.*, 1991; Patel & Fisher, 1993; Sullivan *et al.* 1993). In several resistant cell lines, quantitative reduction of topoisomerase II expression has been correlated with the level of drug resistance in the absence of alterations of drug transport and may represent the major or sole determinant for resistance (Per *et al.*, 1987; Cole *et al.*, 1991; Long *et al.*, 1991; Takano *et al.*, 1991; Webb *et al.*, 1991). In other cell lines selected for resistance in the presence of VP-16, VM-26, anthracyclines or mitoxantrone, there is not only reduced topoisomerase II expression but also reduced drug accumulation, often (but not always) correlated with amplification of the *mdr1* gene and overexpression of the 150-180 kDa P-glycoprotein drug efflux pump (Ferguson *et al.*, 1988; Deffie *et al.*, 1989; Matsuo *et al.*, 1990; Politi *et al.*, 1990; Friche *et al.*, 1991; Long *et al.*, 1991; Kamath *et al.*, 1992; de Jong *et al.*, 1993). Qualitative alterations in topoisomerase II activity have also been reported (Danks *et al.*, 1988; Zwelling *et al.*, 1989; Sullivan *et al.*, 1989; 1993) in cell lines selected for resistance in the presence of topoisomerase II inhibitors (Gupta *et al.*, 1983; Beran & Anderson, 1987; Danks *et al.*, 1987; Sullivan *et al.*, 1993) In several of these resistant human leukaemia cell lines point mutations have been identified within or near nucleotide-binding consensus sequences of the topoisomerase II gene (Bugg *et al.*, 1991; Hinds *et al.*, 1991; Lee *et al.*, 1992; Danks *et al.*, 1993; Chan *et al.*, 1993). Point mutations have also been identified near the active-site tyrosine 804 in resistant cell topoisomerase IIα obtained from CCRF CEM cells selected for resistance in the presence of VM-26 or VP-16 (Danks *et al.*, 1993; Patel & Fisher, 1993). In resistant cell lines exhibiting qualitative changes in topoisomerase II activity there are neither quantitative alterations in topoisomerase II expression nor changes in drug accumulation or overexpression of P-glycoprotein. Thus, stable resistance to topoisomerase II inhibitors can be manifest as (1) altered topoisomerase II expression alone or (2) together with the P-glycoprotein multiple drug resistance phenotype or (3) as a result of a mutation(s) resulting in a qualitative change in topoisomerase II activity.

In this paper, we describe a subline of human leukaemia K562 cells selected for resistance in the presence of VP-16 which has no alterations in drug accumulation compared with sensitive K562 cells. These stably resistant K/VP.5 cells have decreased expression of topoisomerase II mRNA and protein compared with parental K562 cells. In addition to this quantitative decrease in topoisomerase II, K/VP.5 cells exhibit qualitative changes in topoisomerase II activity related to stability of drug-induced topoisomerase II-DNA complexes and alterations in nucleotide dependency for formation of topoisomerase II-DNA complexes. Thus, resistance to topoisomerase II inhibitors in this novel resistant cell line is due to both qualitative and quantitative changes at the level of topoisomerase II.

Correspondence: J.C. Yalowich, Department of Pharmacology, University of Pittsburgh School of Medicine, Biomedical Sciences Tower, 13th Floor, Pittsburgh, PA 15261, USA.
Received 16 August 1993; and in revised form 18 November 1993

### Materials and methods

### *Drugs and chemicals*

[U-$^3$H]VP-16 was obtained from Moravek Biochemicals (Brea, CA, USA) and was more than 95% radiochemically pure as measured by high-performance liquid chromatography (Sinkule & Evans, 1984). [2-$^{14}$C]Thymidine (53 mCi mmol$^{-1}$), [U-$^{14}$C]leucine, [methyl-$^3$H]thymidine (20 Ci mmol$^{-1}$) and [α-$^{32}$P]dCTP were obtained from New England Nuclear (Boston, MA, USA). VP-16 and VM-26 were provided by Bristol-Myers Squibb (Wallingford, CT, USA). Amsacrine, adriamycin and mitoxantrone were obtained from the Drug Investigational Branch of the National Cancer Institute. Vincristine, vinblastine, podophyllotoxin, camptothecin and proteinase K were obtained from Sigma (St Louis, MO, USA). Water-insoluble drugs were prepared in dimethylsulphoxide (DMSO) such that solvent concentrations did not exceed 1% in the culture medium or buffer after drug treatment. DMSO was also included in control flasks at equivalent levels. Protein dye reagent and agarose were purchased from BioRad Laboratories (Richmond, CA, USA). L.F. Liu, Johns Hopkins University, Baltimore, MD, USA, generously provided antisera (IID3) against human DNA topoisomerase II as well as the DNA topoisomerase II cDNA probe (ZII69) contained in the plasmid pC15 and the DNA topoisomerase I cDNA probe contained in the plasmid pSH5-13. F.H. Drake, SmithKline Beecham Laboratories (King of Prussia, PA, USA), kindly provided a sample of purified DNA topoisomerase II.

### *Cells, media and incubation conditions*

Human leukaemia K562 cells were grown in suspension culture in Dulbecco's modified Eagle medium (DMEM) plus 10% fetal calf serum and L-glutamine. L1210 cells were grown in DMEM, 10% horse serum and L-glutamine. Resistant K/VP.5 cells were selected by first periodic and then continuous exposure of K562 cells to 0.5 μM VP-16 for 1 year, after which clones were isolated by limiting dilution (Norman & Thompson, 1977). The K/VP.5 clone had a doubling time of 22 h compared with the parental K562 doubling time of 18 h. The resistant clone has been stably resistant in the absence of drug for 2 years. Cytogenetic analysis indicated that both parental and resistant cells were hyperdiploid. No homogeneously staining regions or double minute chromosomes were present in K/VP.5 cells.

### *Drug-induced growth inhibition and drug accumulation*

Log-phase sensitive and resistant cells were adjusted to $1 \times 10^5$ cell ml$^{-1}$ and incubated with various concentrations of a number of drugs for a period of 48 h, after which cells were counted on a model ZBF Coulter counter (Coulter Electronics, Hialeah, FL, USA). The extent of growth (beyond the starting concentration of $1 \times 10^5$ cells ml$^{-1}$) in drug-treated *vs* control cells was ultimately expressed as per cent inhibition of control growth. The 50% growth-inhibitory concentration for each drug in each cell line was calculated from replicate dose–response curves generated from separate experiments.

For drug accumulation studies, K562 and K/VP.5 cells were suspended in a pH 7.4 buffer (buffer L) of 110 mM sodium chloride, 5 mM potassium chloride, 1 mM magnesium chloride, 5 mM sodium dihydrogen phosphate, 25 mM 4-(2-hydroxy-ethyl)-1-piperazineethanesulphonic acid and 10 mM glucose, at a final concentration of $0.5 \times 10^7$ cells ml$^{-1}$. Cells were stirred in specially designed flasks by revolving Teflon paddles in a 37°C water bath, as described previously (Yalowich, 1987). One millilitre portions of cell suspension containing [$^3$H]VP-16 were periodically injected into ten volumes of 0.85% sodium chloride solution at 0°C. Cell fractions were then separated by centrifugation and washed twice with 0.85% sodium chloride at 0°C. The washed pellet was drawn up into a plastic pipette tip, extruded onto a polyethylene tare and dried overnight at 70°C. The dried pellets were weighed, placed in a glass scintillation vial and dissolved in 0.25 ml of 1 M potassium hydroxide for 90 min at 70°C. The digest was neutralised with 0.25 ml of 1 M hydrochloric acid; 4 ml of aqueous counting scintillant (Amersham Corp., Arlington Heights, IL, USA) was added, and radioactivity was determined by liquid scintillation counting. Results yield cellular drug content expressed as nmol per g dry weight. Intracellular water per g dry weight was determined from the difference between the wet and dry weights of cell pellets minus the [$^{14}$C]inulin space, as described elsewhere (Yalowich & Goldman, 1984). Molar intracellular drug concentration was then determined from the molar content of cell VP-16 and the intracellular water volume.

### *DNA damage assays*

For DNA damage experiments, K562 and K/VP.5 cells were labelled for 48 h with [2-$^{14}$C]thymidine (0.02 μCi ml$^{-1}$). L1210 cells were labelled for 16 h with [methyl-$^3$H]thymidine (0.1 μCi ml$^{-1}$). Unlabelled thymidine was added to allow for a final thymidine concentration of 1 μM in the culture medium.

Isolated nuclei were prepared by washing $^{14}$C-labelled whole cells in an ice-cold buffer A (pH 6.4) containing 1 mM potassium dihydrogen phosphate, 5 mM magnesium chloride, 150 mM sodium chloride and 1 mM EGTA (Filipski & Kohn, 1982). The cells were resuspended in 1 ml of this buffer, and an additional 9 ml of buffer A containing 0.3% Triton X-100 was added to lyse the cells. After incubation on ice for 30 min, 40 ml of buffer A was added, and nuclei were pelleted by centrifugation at 1,000 r.p.m. for 10 min in an IEC model HN-SII tabletop centrifuge. Nuclei density was adjusted to $1 \times 10^6$ ml$^{-1}$ in cold buffer. After warming at 37°C for 15 min, the nuclei were treated with VP-16 and other agents in the presence or absence of 1 mM ATP and processed for measurement of DNA damage as described below.

Drug-mediated DNA damage was assessed using the alkaline elution technique for high-frequency single-strand breaks (Kohn *et al.*, 1976). Intact K562 and K/VP.5 cells previously labelled with [2-$^{14}$C]thymidine were suspended at $5 \times 10^5$ cells ml$^{-1}$ in buffer A. These cells were incubated with various concentrations of VP-16 and other agents for 30 min at 37°C. L1210 cells ($5 \times 10^5$) containing [$^3$H]DNA which received 1,500 rad irradiation were added as internal standards to $7.5 \times 10^5$ drug-treated K562 cells containing [$^{14}$C]DNA. After two washings in cold buffer A, cells were layered onto a polyvinyl chloride filter (pore size 0.8 μm; Gelman Sciences, Ann Arbor, MI, USA) and lysed with a solution of 2% sodium dodecyl sulphate, 10 mM disodium EDTA and 0.5 mg ml$^{-1}$ proteinase K. The DNA was eluted from the filter with tetrapropylammonium hydroxide, pH 12.1. The elution flow rate was 0.16 ml min$^{-1}$, with a fractional interval of 5 min. Cells containing [$^3$H]DNA were irradiated on ice with a $^{137}$Cs source (Mark Irradiator; J.L. Sheppard and Associates, Glendale, CA, USA). The frequency of VP-16- and other drug-induced DNA single-strand breaks (SSBs) was quantitated as the fraction of [$^{14}$C]DNA remaining on the filter when either 60% or 75% of the $^3$H-labelled internal standard DNA remains. A calibration curve for relating the frequency of VP-16-induced DNA SSBs to a corresponding effect of radiation (radiation equivalent DNA damage) using $^{14}$C-labelled cells was obtained by plotting rads *vs* [$^{14}$C]DNA retention at 60% or 75% retention of the [$^3$H]DNA internal standard.

Determination of DNA–protein cross-link (DPC) frequency was accomplished as previously described (Ross *et al.*, 1979). Aliquots of [$^{14}$C]thymidine-labelled K562 and K/VP.5 cells ($5 \times 10^5$ ml$^{-1}$) or nuclei ($1 \times 10^6$ ml$^{-1}$), drug-treated or controls, were irradiated on ice with 3,000 rad prior to elution, as were internal control $^3$H-labelled L1210 cells. Cells or nuclei were layered onto polyvinyl-chloride–acrylic co-polymer filters (Metricel DM-800; Gelman Sciences) along with $7.5 \times 10^5$ internal control cells, and lysed with 5 ml of a solution of 0.2% Sarkosyl, 2 M sodium chloride, 0.04 M disodium EDTA, pH 10, which was allowed to flow through the filters by gravity, as was a 3 ml addition of 0.04 M disodium EDTA, pH 10. DNA was eluted with tetrapropylammonium hydroxide, pH 12.1, at a flow rate of 0.035 ml $min^{-1}$. Fractions were collected at 90 min intervals for 12 h. DPC frequencies were calculated according to the bound-to-one terminus model (Ross *et al.*, 1979):

$$\text{DPC (rad equivalents)} = [(1-r)^{-1} - (1-r_o)^{-1}]P_B$$

where $r_o$ and $r$ are the fractional filter retentions of DNA extrapolated to zero time from [$^{14}$C]thymidine-labelled control and drug-treated cells (or nuclei) respectively and $P_B$ is the radiation dose administered (3,000 rad). The greater the DNA retention of drug-treated cells relative to controls, the greater the DPC frequency.

### *DNA topoisomerase II–DNA covalent complex formation assay–intact cells and nuclei*

Mid-log cells (1.5–2.0 × $10^5$ cells $ml^{-1}$) were labelled overnight with 0.5 μCi $ml^{-1}$ [methyl-$^3$H]thymidine (0.5 Ci $mmol^{-1}$) and 0.1 μCi $ml^{-1}$ [U-$^{14}$C]leucine (318 mCi $mmol^{-1}$) in DMEM containing 5% FBS. Cells were then pelleted and resuspended in fresh DMEM/5% FBS and incubated for 1 h at 37°C. For experiments measuring the stability of topoisomerase II–DNA covalent complexes after VP-16 removal, cells were washed, resuspended in buffer L and equilibrated to 37°C. VP-16 was added to 20–200 μM and incubation continued for an additional 15 min at 37°C. Cells were pelleted for 90 s at 2,400 *g*, washed with ice-cold buffer L and repelleted. Cells were resuspended to 1 × $10^6$ $ml^{-1}$ in L buffer at 37°C. At selected intervals, 1 × $10^6$ cells were removed and added to 10 ml of ice-cold PBS and held on ice until all time points were collected. Cells were pelleted, lysed, cellular DNA sheared and protein–DNA complexes precipitated with SDS and potassium chloride as described by Zwelling *et al.* (1989). For experiments examining the effect of ATP on VP-16-induced stabilisation of topoisomerase II–DNA cleavable complexes, nuclei were isolated from cells as described above and adjusted to 2 × $10^6$ nuclei $ml^{-1}$ in buffer A (pH 7.4) containing 0.5 mM ATP. After prewarming at 37°C for 5 min, 200 μM VP-16 or 0.4% DMSO (control) was added and incubation continued at 37°C for an additional 15 min. Nuclei were pelleted, lysed, cellular DNA sheared and complexes precipitated with SDS–potassium chloride exactly as for whole cells.

### *Topoisomerase II catalytic activity*

Topoisomerase II-containing extracts of nuclei were prepared from 1–2 × $10^8$ K562 and K/VP.5 cells as previously described (Danks *et al.*, 1988). When the aqueous volume contributed by the nuclei is taken into consideration, the final sodium chloride concentration of the nuclear extracts varied between preparations from 0.7 to 0.85 M. *Crithidia fasiculata* was labelled with 8 μCi $ml^{-1}$ [methyl-$^3$H]thymidine and kinetoplast mitochondrial DNA (kDNA) isolated as previously described (Sahai & Kaplan, 1986). Topoisomerase II catalytic activity was measured by decatenation of kDNA (Sullivan *et al.*, 1989). Each 40 μl assay contained 50 mM Tris (pH 7.5), 85 mM potassium chloride, 10 mM magnesium chloride, 0.5 mM DTT, 0.5 mM disodium EDTA, 30 μg $ml^{-1}$ BSA, 0–1 mM ATP, 0–100 μM VP-16 (in DMSO), 1 μg (10,000 c.p.m.) of $^3$H-labelled kDNA and 0–3 μg of nuclear extract topoisomerase II from K562 or K/VP.5 cells. After incubation at 30°C for 30 min, reactions were stopped by addition of 10 μl of 2.5% SDS and were then centrifuged for 15 min at 8,000 *g* at 25°C. Duplicate 10 μl samples from each tube were counted in a liquid scintillation spectrometer in 3.5 ml of Ecolite (ICN Biochemicals, Irvine, CA, USA). Decatenation was quantitated subsequent to subtraction of counts found in DMSO controls in the absence of nuclear extract topoisomerase II.

### *Western blots of cell lysates*

Lysates from 2–5 × $10^6$ K562 or K/VP.5 cells were prepared by the addition of an equal volume of 2 × gel loading buffer (0.1 M Tris–HCl, pH 6.8, 20% glycerol, 2% SDS, 0.5 M β-mercaptoethanol). Lysates were boiled for 5 min, sonicated to reduce viscosity and protein content determined using the BioRad protein assay. Samples containing 10 μg were electrophoresed through 6% SDS–polyacrylamide gel (Laemmli, 1970) and electroblotted at 400 mA overnight to nitrocellulose (Towbin *et al.*, 1979) using a Hoefer (San Franscisco, CA, USA) Transfor apparatus. Blots were incubated with human topoisomerase II-specific rabbit antiserum, IID3 (from L.F. Liu), affinity-purified topoisomerase IIα-specific antibody FHD22 (Webb *et al.*, 1993) or affinity-purified topoisomerase IIβ-specific antibody FHD21 (Chung *et al.*, 1989). The affinity-purified, isoform-specific antibodies were provided by F.H. Drake (SmithKline Beecham). Bound antibodies were detected using alkaline phosphatase-conjugated goat anti-rabbit IgG and the ProtoBlot Western Blot AP System of Promega Biotech (Madison, WI, USA). Levels of topoisomerase II were quantitated by scanning positive films of photographed blots using a Visage 110 image analyser (Ann Arbor, MI, USA).

### *Northern blot analysis of cellular RNAs*

Total cellular RNA was isolated from 1–2 × $10^8$ K562 and K/VP.5 cells (Chomczynski & Sacchi, 1987). RNA (10 μg) was electrophoresed through 1.2% agarose gels containing 0.3 M formaldehyde, 40 mM 3-(*N*-morpholono)propane sulphonic acid (MOPS) pH 7.0, 10 mM sodium acetate trihydrate, 1 mM EDTA, transferred to Nytran (Schleicher & Schuell, Keene, NH, USA) by capillary blotting overnight, baked for 2 h at 80°C and hybridised using standard techniques (Maniatis *et al.*, 1982). Specific RNAs were detected by autoradiography at − 70°C using intensifying screens and Kodak XAR-5 X-ray film.

### *Probe synthesis and labelling*

The human topoisomerase IIα cDNA probe (ZII69) contained in the plasmid pC15 was provided by L.F. Liu. A human topoisomerase IIβ cDNA probe, synthesised by polymerase chain reaction (PCR) amplification using topoisomerase IIβ-specific primers, was provided by D.P. Suttle (St Jude Children's Research Hospital, Memphis, TN, USA). The $\beta_2$-microglobulin cDNA probe in plasmid p$\beta_2$m was provided by K.B. Tan. Cloned inserts were removed from plasmids pC15 and p$\beta_2$m by digestion with restriction enzymes and isolation on agarose gels. cDNA fragments were used as templates for probe synthesis using Klenow fragment, [α-$^{32}$P]dCTP (3,000 Ci $mmol^{-1}$) and random primer initiation (Feinberg & Vogelstein, 1983).

### Results

### *Drug accumulation and growth-inhibitory effects in sensitive and resistant cells*

The K/VP.5 subline is 30-fold resistant to the 48 h growth-inhibitory effects of the selecting agent VP-16, and equally cross-resistant to VM-26 (Table I). In addition, there was lower (5- to 13-fold) cross-resistance to *m*-AMSA, adriamycin and mitoxantrone, three other agents that are known to inhibit topoisomerase II. K/VP.5 cells cultured in the absence of VP-16 have maintained this level of resistance for 2 years. In contrast, K/VP.5 cells were not cross-resistant to the microtubule inhibitors vincristine, vinblastine and podophyllotoxin, the antimetabolite arabinosyl cytosine or to the DNA topoisomerase I inhibitor camptothecin (Table I). Figure 1 indicates that the steady-state concentrations of VP-16 in K/VP.5 cells were slightly higher than in parental K562 cells when cells were exposed to 2.5–20 μM [$^3$H]VP-16.

Table I Cross-resistance of K/VP.5 cells to anti-cancer agents

| Anti-cancer agent | $IC_{50}$ values (nM)[a] K562 | | K/VP.5 | | Relative resistance[b] |
|---|---|---|---|---|---|
| VP-16 | 121 ± 48 | (21)[c] | 3640 ± 1617 | (21) | 30 |
| VM-26 | 10.3 ± 0.5 | (4) | 369 ± 63 | (4) | 36 |
| Amsacrine | 41.4 ± 22.3 | (8) | 524 ± 240 | (8) | 12.7 |
| Adriamycin | 38.6 ± 10.8 | (7) | 178 ± 71 | (8) | 4.8 |
| Mitoxantrone | 14.7 ± 4.8 | (5) | 66.0 ± 21.4 | (5) | 4.5 |
| Camptothecin | 5.2 ± 0.9 | (4) | 5.9 ± 1.5 | (4) | 1.1 |
| Vinblastine | 8.2 ± 5.2 | (9) | 5.7 ± 2.8 | (13) | 0.7 |
| Vincristine | 5.8 ± 2.7 | (7) | 4.1 ± 1.1 | (5) | 0.7 |
| Podophyllotoxin | 7.6 ± 7.9 | (7) | 9.1 ± 4.4 | (4) | 1.2 |
| ARA-C | 4.7 ± 0.2 | (3) | 6.8 ± 1.5 | (3) | 1.4 |

[a]50% inhibitory concentration in a 48 h growth inhibition assay. [b]$IC_{50}$ of K/VP.5 cells divided by that of the parental K562 cell line. [c]Mean ± s.d.; numbers in parentheses, numbers of experiments run on different days.

**Figure 1** Steady-state cellular concentrations of VP-16 in K562 (●) and K/VP.5 (■) cells exposed to 2.5–20 μM [$^3$H]VP-16. Steady-state concentrations of VP-16 were achieved within 10–15 min, regardless of extracellular VP-16 concentration. For each experiment, 3–5 measurements at steady state were obtained at each drug concentration. Points, mean of three separate experiments; bars, s.d.

These results are consistent with those previously reported (Beran & Anderson, 1987), in which accumulation of *m*-AMSA was slightly increased in HL-60 cells selected for resistance in the presence of *m*-AMSA. Accounting for intracellular water content (Yalowich & Goldman, 1984), which was 5.19 ± 0.74 and 5.37 ± 0.13 ml $g^{-1}$ dry weight for K/VP.5 and K562 cells respectively, the intracellular VP-16 concentration was 22.2 μM and 30.2 μm for K562 and K/VP.5 cells respectively, when the intracellular VP-16 concentration was 20 μM. These results demonstrate that VP-16 does not concentrate extensively within cells and suggests that decreased drug accumulation does not play a role in resistance to VP-16 in K/VP.5 cells. In addition, averaging results from 4–7 separate experiments, there were no significant differences in VP-16 unidirectional efflux in sensitive and resistant cells loaded to steady-state drug concentrations in the presence of 5 μM [$^3$H]VP-16 ($t_{1/2}$ = 103 ± 12 s and 108 ± 2 s for K562 and K/VP.5 cells respectively) using methods previously reported (Yalowich, 1987). Finally, using the C219 monoclonal antibody, there was no overexpression of P-glycoprotein (180 kDa) in cell membranes from K/VP.5 compared with K562 cells (M. Meyers & J.C. Yalowich, unpublished data).

*Drug-induced DNA single-strand breaks and DNA-protein cross-linking*

DNA single-strand break frequency was reduced in K/VP.5 cells compared with parental K562 cells when these two lines were incubated with varying concentrations of VP-16 (Figure 2). A similar reduction in single-strand breaks was observed in resistant cells in the presence of *m*-AMSA and adriamycin (not shown). VP-16 (10–100 μM)-induced single-strand breaks were also reduced in isolated K/VP.5 nuclei compared with K562 nuclei (results not shown). In three separate experiments in which nuclei were incubated with 10 μM *m*-AMSA, the single-strand break frequency was 1857 ± 238 and 671 ± 46 radiation equivalents (mean ± s.e.) for K562 and K/VP.5 nuclei respectively. When the DNA topoisomerase I inhibitor camptothecin (0.1–1.0 μM) was added to isolated nuclei from K562 and K/VP.5 cells, there was no difference in drug-induced DNA strand break frequency (not shown). Finally, DNA-protein cross-linking (DPC) induced by VP-16, VM-26 and *m*-AMSA was decreased in K/VP.5 compared with K562 cells (Figure 3).

*Topoisomerase II catalytic activity*

Decatenation activity of nuclear extract topoisomerase II (0–2 μg) isolated from K562 and K/VP.5 cells indicated that topoisomerase II catalytic activity was reduced 5- to 7-fold in resistant compared with sensitive cells as measured by the amount of protein required for 50% decatenation (Figure 4). Since decreased topoisomerase II levels could account for reduced topoisomerase II catalytic activity, the next set of experiments examined topoisomerase II expression in K562 and K/VP.5 cells.

*Topoisomerase II levels: Western blot analysis*

The amount of topoisomerase II in whole-cell lysates and in 0.8 M sodium chloride nuclear extracts from K562 and K/VP.5 cells was determined by Western blotting utilising rabbit IID3 antiserum to human DNA topoisomerase II (Figure 5). Two bands were observed in the lane containing purified topoisomerase II from P388 leukaemia cells (Drake *et al.*, 1987) as well as in lanes corresponding to K/VP.5 nuclear extracts. The molecular weights of these bands were 170 ± 3 kDa and 179 ± 3 kDa (mean ± s.e.; $n = 5$), corresponding to previously characterised topoisomerase IIα and topoisomerase IIβ isoforms of this enzyme respectively (Chung *et al.*, 1989; Drake *et al.*, 1989). A pronounced reduction in the level of topoisomerase II in the lower molecular weight band (topoisomerase IIα) in both whole-cell lysates and nuclear extracts was observed in resistant K/VP.5 cells. Levels of topoisomerase IIα were quantitated by densitometric scanning of immunolabelled 170 kDa bands from whole-cell lysates or nuclear extracts. The topoisomerase II signals (area under scanned peak) obtained with various amounts of K562 protein served as a standard curve from which signals for K/VP.5 were quantitated. Using whole-cell lysates, we observed a 7.8- ± 2.3-fold reduction in the level of topoisomerase II ($M_r$ 170,000) in K/VP.5 compared with K562 cells (mean ± s.e., from three separate lysate preparations). In nuclear extracts there was a 4.5-fold decrease in the level of topoisomerase II in K/VP.5 compared with K562 cells (not shown). The higher molecular weight band (topoisomerase IIβ) showed much less staining in both sensitive and resistant cell lysates and nuclear extracts and could not be accurately quantitated. However, we independently quantitated levels of each topoisomerase II isoform by probing Western blots (not shown) of whole-cell lysates with polyclonal antibodies that specifically recognise either topoisomerase IIα (antibody FHD22) or topoisomerase IIβ (antibody FHD21). Results from three independent blots showed that topoisomerase IIα and topoisomerase IIβ were reduced 5.6- ± 1.6-fold and 2.7- ± 0.1-fold respectively (mean ± s.e.) in K/VP.5 compared with K562 cells.

**Figure 2** DNA single-strand breaks in K562 (○) and K/VP.5 (●) cells treated for 30 min in the presence of various concentrations of VP-16. A calibration curve for relating the frequency of VP-16-induced single-strand breaks to a corresponding effect of radiation (radiation equivalent DNA damage) was obtained by plotting rads *vs* [$^{14}$C]DNA retention at 75% retention of the [$^{3}$H]DNA internal standard. Points, mean of 5–10 separate experiments; bars, s.e.

### *Topoisomerase II expression: Northern blot analysis of total RNA*

Using the human topoisomerase II cDNA probe (ZII69) and total cellular RNA isolated from K562 and K/VP.5, we observed a 2.9-fold reduction in expression of 6.2 kb topoisomerase IIα RNA in K/VP.5 compared with parental K562 cells (Figure 6). Co-hybridisation of a $\beta_2$-microglobulin ($\beta_2$ m) cDNA to the 1.0 kb $\beta_2$ m RNA served as an internal control for variable loading of RNA. Relative levels of topoisomerase II mRNA were quantitated by densitometric scanning of autoradiographs and corrected for $\beta_2$ m levels. Similar results have been obtained with four different RNA preparations from K562 and K/VP.5 cells. There was a similar 2.9-fold decrease in expression of topoisomerase IIβ mRNA in resistant compared with sensitive cells (Figure 7). In contrast, there were no differences in expression of DNA topoisomerase I mRNA in K562 and K/VP.5 cells (not shown).

### *Reversal of VP-16-induced DNA damage*

The rate of reversal of VP-16-induced single-strand break damage was examined subsequent to a 30 min incubation of K562 cells with 5 μM VP-16 and of K/VP.5 cells with 40 μM VP-16 in order to yield similar initial strand break frequencies in both cell lines. Averaging results from five experiments the initial DNA strand break frequency was 1436 ± 151 and 1130 ± 150 radiation equivalents for K562 and K/VP.5 cells respectively (mean ± s.e.). After suspension of cells in drug-free buffer at least seven determinations of remaining DNA strand breaks were made during the next 40 min (results not shown). The reversal of VP-16-induced DNA damage was more rapid in resistant K/VP.5 cells compared with sensitive K562 cells; averaging five separate paired experiments the $t_{1/2}$ for reversal of DNA damage was 9.7 ± 1.5 and 16.1 ± 1.9 min respectively (mean ± s.e.; $P = 0.002$, Student's paired

**Figure 3** DNA–protein cross-linking produced by VP-16, VM-26, and mAMSA in K562 (open bars) and K/VP.5 (shaded bars) cells. Cells were treated with drugs at 37°C for 30 min at the indicated concentrations. DNA–protein cross-linking was quantitated by alkaline elution from polyvinyl chloride filters (0.8 μm) and expressed as radiation equivalents (see Materials and methods). Columns, mean of six separate experiements; bars, s.e.

**Figure 4** Decatenation of kinetoplast DNA as a function of nuclear extract topoisomerase II. Salt (0.8 M)-extracted topoisomerase II from the nuclei of K562 and K/VP.5 cells was incubated with 1 μg of $^{3}$H-labelled kinetoplast DNA in the presence of 1 mM ATP for 30 min at 30°C. Decatenation of DNA was measured subsequent to centrifugal separation of catenated from decatenated DNA as described in Materials and methods.

$t$-test). Since the efflux rate of VP-16 from K562 and K/VP.5 cells does not differ ($t_{1/2} \approx 2$ min for both lines, see above), and since VP-16 has been considered a non-intercalating drug that does not bind directly to DNA, the more rapid reversal of VP-16-induced DNA damage in K/VP.5 cells compared with K562 cells suggests that resistant cells contain an altered binding site(s) for VP-16: either an altered form of topoisomerase II and/or an altered modulator of topoisomerase II activity. In addition, VP-16 efflux from both cell lines ($t_{1/2} \approx 2$ min) is more rapid than the rate of DNA damage reversal from either cell line ($t_{1/2} = 9.7-16.1$ min), indicating that VP-16 transport is not rate limiting to the process of reversal of VP-16-induced DNA damage. Hence, these data suggest that altered stability of VP-16-induced topoisomerase II–DNA covalent complexes in resistant cells leads to more rapid reversal of DNA damage.

The specificity of VP-16-induced DNA damage and its more rapid reversal in K/VP.5 compared with K562 cells was revealed by experiments in which equivalent single-strand breaks were introduced into the DNA of K562 and K/VP.5 cells by exposure of cells at 4°C to 1,500 rad of gamma-irradiation. There was no difference in the rate of reversal of DNA damage when cells were warmed to 37°C. In three experiments the $t_{1/2}$ for reversal of radiation-induced DNA damage was $11.1 \pm 1.7$ and $10.1 \pm 2.2$ min (mean ± s.e.) in K562 and K/VP.5 cells respectively. These results again are consistent with the idea that VP-16-induced DNA damage and its more rapid reversal in K/VP.5 cells is determined by interaction with an intracellular target that is altered in these resistant cells.

**Figure 5** Topoisomerase II protein levels from K562 and K/VP.5 cells. PBS-washed cells were lysed, sonicated, electrophoresed through SDS–polyacrylamide gels, and electroblotted to nitrocellulose. The blot was labelled first with topoisomerase II-specific polyclonal antisera then alkaline phosphatase-conjugated goat anti-rabbit antibody as described in Materials and methods. Shown are duplicate loadings of 10 μg of protein from lysates of K562 and K/VP.5 cells (corresponding to lysates from *c.* $1.5 \times 10^5$ cells). The last lane contains purified topoisomerase II from P388 cells. Numbers to the right of the figures are molecular size markers (in kDa).

### *Stability of VP-16-induced topoisomerase II–DNA complexes*

The stability of VP-16-induced topoisomerase II–DNA complexes was measured subsequent to a 15 min incubation of K562 cells with 20 μM VP-16 and of K/VP.5 cells with 200 μM VP-16. At these VP-16 concentrations, the steady-state level of topoisomerase II–DNA complex was similar (within 15%) in sensitive *vs* resistant cells. After suspension of cells in drug-free buffer, there was a more rapid dissociation of the topoisomerase II–DNA complex in resistant compared with sensitive cells (Figure 8). In eight separate paired experiments, the half-life for reversal of covalent complexes of topoisomerase II–DNA in K/VP.5 cells averaged $6.2 \pm 0.3$ min as compared with $9.0 \pm 0.6$ min for K562 cells (mean ± s.e.; $P = 0.003$, Student's paired $t$-test). Thus, a significant reduction in stability of VP-16-induced topoisomerase II binding to DNA may be a factor in decreased DNA damage and/or a more rapid rate of recovery from DNA damage in resistant K/VP.5 cells.

**Figure 6** Topoisomerase IIα mRNA levels in K562 and K/VP.5 cells. RNA was purified from mid-log phase cells and 10 μg was electrophoresed through formaldehyde-containing agarose gels. After transfer to a nylon membrane, RNAs were hybridised to $^{32}$P-labelled topoisomerase IIα and $β_2$-microglobulin cDNA probes, and autoradiographed as described in Materials and methods. Topoisomerase IIα mRNA signals were corrected for $β_2$-microglobulin levels. Averaging results from three separate blots, there was a 2.91- ± 0.95-fold decrease in topoisomerase IIα mRNA in K/VP.5 compared with K562 cells (mean ± s.d.; $P < 0.02$, Wilcoxon's signed-ranks test).

**Figure 7** Topoisomerase IIβ mRNA levels in K562 and K/VP.5 cells. RNA was purified, electrophoresed, immobilised and hybridised to $^{32}$P-labelled topoisomerase IIβ and $β_2$-microglobulin cDNA probes, and autoradiographed exactly as for Figure 7. Topoisomerase IIβ mRNA signals were corrected for $β_2$-microglobulin levels. Averaging results from three separate blots, there was a 2.87- ± 0.62-fold decrease in topoisomerase IIβ mRNA in K/VP.5 compared with K562 cells (mean ± s.d.; $P < 0.01$, Wilcoxon's signed-ranks test).

**Table II** Effects of ATP (1 mM) on VP-16-induced single-strand break frequency and DNA-protein cross-linking in sensitive K562 and resistant K/VP.5 nuclei

| *Type of DNA lesion* | *Experimental condition* | *Radiation equivalents*[a] *K562 (25 μM VP-16)* | | | *K/VP.5 (100 μM VP-16)* | | |
|---|---|---|---|---|---|---|---|
| Single-strand | Control | 476 ± 46 | (9)[b] | | 442 ± 30 | (9) | |
| breaks (SSBs) | + ATP | 1322 ± 86 | (11) | [277][c] | 770 ± 37 | (4) | [174][c] |
| DNA-protein | Control | 497 ± 28 | (5) | | 301 ± 47 | (5) | |
| cross-links (DPCs) | + ATP | 1136 ± 96 | (5) | [229][d] | 523 ± 83 | (5) | [174][d] |

[a]Nuclei were incubated with the indicated concentrations of VP-16 for 30 min in the presence or absence of 1 mM ATP, following which alkaline elution methodology was used for quantitation of radiation equivalent SSBs and DPCs as described in Materials and methods. [b]Data are expressed as mean ± s.e. Numbers in parentheses indicate the number of individual experiments run on different days. [c]Numbers in brackets represent the percentage increase in SSBs in the presence of ATP. The percentage increase in K562 nuclei is significantly greater than in K/VP.5 nuclei ($P = 0.0001$, Student's $t$-test adjusted for non-equal sample variances). [d]Numbers in brackets represent the percentage increase in DPCs in the presence of ATP. The percentage increase in K562 nuclei is significantly greater than in K/VP.5 nuclei ($P = 0.039$, Student's paired $t$-test).

**Figure 8** Stability of topoisomerase II-DNA covalent complexes in VP-16-treated K562 and K/VP.5 cells. Cells were prelabelled with [methyl-$^3$H]thymidine and [U-$^{14}$C]leucine, and exposed to 20 μM VP-16 (K562) or 200 μM VP-16 (K/VP.5) for 15 min. Cells were washed free of drug, and at the indicated time points (10 s to 30 min) aliquots were removed and potassium chloride-SDS-precipitable complexes isolated as described in Materials and methods. Results were normalised to $^{14}$C counts as an internal standard for cell number, and are expressed relative to potassium chloride-SDS-precipitable $^3$H counts recovered at the end of VP-16 treatment (0 min).

**Figure 9** Stimulation of topoisomerase II-DNA covalent complexes by ATP in nuclei isolated from VP-16-treated K562 and K/VP.5 cells. Nuclei, from cells prelabelled with [methyl-$^3$H]thymidine and [U-$^{14}$C]leucine, were incubated for 15 min in the presence of 200 μM VP-16 and various concentrations of ATP. Potassium chloride-SDS-precipitable complexes were isolated and the $^3$H-counts normalised to cell number using $^{14}$C as an internal standard for cell number as described in Materials and methods. Results are expressed as fold increase relative to complexes isolated from cells incubated in the absence of VP-16 and ATP.

### *Nucleotide-dependent VP-16-induced formation of single-strand breaks, DNA-protein cross-links and topoisomerase II-DNA complexes*

ATP (1 mM) stimulates VP-16 (25 μM)-induced SSBs almost 3-fold in nuclei isolated from K562 cells (Table II). In contrast, when the VP-16 concentration was increased to 100 μM to achieve a similar SSB frequency in K/VP.5 nuclei, ATP enhanced drug-induced DNA damage less than 2-fold. Similarly, ATP-mediated enhancement of VP-16-induced DNA-protein cross-linking was significantly less in resistant K/VP.5 than in sensitive K562 nuclei (Table II). Since topoisomerase II catalytic activity is dependent on binding (but not hydrolysis) of ATP (Osheroff, 1989), and since ATP is known to stimulate VP-16-, *m*-AMSA-, ellipticine- and 5-iminodaunorubicin-induced DNA damage in isolated nuclei from L1210 cells (Glisson *et al.*, 1984; Pommier *et al.*, 1984), our results are consistent with the hypothesis that there is an altered functional interaction of ATP with topoisomerase II in resistant K/VP.5 cells. ATP stimulation of VP-16 (200 μM)-induced topoisomerase II-DNA covalent complexes was less in K/VP.5 than in K562 nuclei (Figure 9). At 1 mM ATP, covalent complex formation was increased 8-fold for K562 cells but only 2-fold for K/VP.5 cells. In addition, using a non-hydrolysable form of ATP, 5′-adenylyl-imidodiphosphate (1 mM), there was less stimulation of VP-16-induced topoisomerase II-DNA covalent complexes in K/VP.5 nuclei (1.7- ± 0.2-fold) than in K562 (3.2- ± 0.4-fold) nuclei (mean ± s.e., $P < 0.05$; Student's paired $t$-test; data not shown from four separate experiments). These data further support a qualitative alteration in resistant cell topoisomerase II affecting nucleotide-dependent VP-16-induced stabilisation of topoisomerase II-DNA complexes.

### *Topoisomerase II catalytic activity*

After normalising for the difference in topoisomerase II protein in nuclear extracts obtained from sensitive and resistant cells, 2-fold greater ATP concentration was required for 50% decatenation of $^3$H-labelled kinetoplast DNA using nuclear extract topoisomerase II from K/VP.5 compared with K562 cells (Figure 10). When ATP concentration was fixed at 1 mM, there was no significant difference in VP-16-induced

**Figure 10** Effects of ATP on nuclear extract topoisomerase II decatenation of kinetoplast DNA. Sodium chloride (0.8 M)-extracted topoisomerase II from the nuclei of K562 and K/VP.5 cells was incubated with 1 μg of $^3$H-labelled kinetoplast DNA in the presence of 0–1 mM ATP for 30 min at 30°C. Decatenation of kinetoplast DNA was measured subsequent to centrifugal separation of catenated from decatenated DNA as described in Materials and methods. In order to normalise for the 5-fold differences in topoisomerase II levels in nuclear extracts from these two cell lines, 0.36 μg and 1.8 μg of nuclear extract protein was used for K562 and K/VP.5 cells respectively. Points represent the mean for 3–6 separate experiments; bars, s.e.

**Figure 11** Inhibition of topoisomerase II catalytic decatenation activity by VP-16. Nuclear extract topoisomerase II from the nuclei of K562 and K/VP.5 cells was incubated with 1 μg of $^3$H-labelled kineteoplast DNA in the presence of 1 mM ATP and 0–100 μM VP-16 for 30 min at 30°C. Decatenation of kinetoplast DNA was measured as described in Figure 10 and Materials and methods. Nuclear extract protein content was normalised for differences in topoisomerase II content in K562 and K/VP.5 cells. Inhibition of decatenation is expressed relative to decatenation activity observed in the absence of VP-16. Points represent the mean of four separate experiments; bars, s.e. The 50% inhibitory concentrations were 22.0 ± 1.7 and 29.3 ± 4.1 μM VP-16 for K562 and K/VP.5 cells respectively ($P = 0.21$).

inhibition of catalytic decatentation using nuclear extract preparations from sensitive and resistant cells (Figure 11).

### Discussion

The results presented in this study indicate that resistance to VP-16 and cross-resistance to other topoisomerase II inhibitors in K/VP.5 cells is associated with alterations in both the levels and the drug-induced DNA-binding activity of topoisomerase II. Drug-induced DNA strand breaks, DNA–protein cross-links and topoisomerase II catalytic activity were reduced in K/VP.5 compared with K562 cells and nuclei (Figures 2–4); these results correlate with the reduction in levels of topoisomerase II protein (Figure 5) and parallel the reduced levels of topoisomerase II mRNA (Figure 6) in resistant compared with sensitive cells. Together these results indicate a quantitative reduction in topoisomerase II expression in K/VP.5 cells. Previously, we performed topoisomerase II immunoblot depletion experiments which demonstrated that topoisomerase II from resistant cells was not bound to DNA at VP-16 concentrations which did induce topoisomerase II–DNA binding in sensitive cells (Ritke & Yalowich, 1993). Similar topoisomerase II immunoblot depletion experiments demonstrated qualitative changes in topoisomerase IIα in a VP-16-resistant small-cell lung cancer cell line which also exhibited quantitative topoisomerase IIα alterations (Mirski et al., 1993). Together, these results suggested qualitative alterations in topoisomerase II in resistant K/VP.5 cells.

In order to better characterise qualitative alterations in resistant cell topoisomerase II function in the present study, we have used different VP-16 concentrations to achieve similar levels of DNA damage or topoisomerase II–DNA binding in sensitive vs resistant cells. In this manner we have 'normalised' for differences in topoisomerase II protein in K562 and K/VP.5 cells. Under these experimental conditions, the more rapid reversal of drug-induced DNA damage in resistant K/VP.5 cells compared with K562 cells and the attenuated effect of ATP on the stimulation of VP-16-induced DNA strand breaks and DPC in nuclei from resistant cells (Table II) strongly suggests that, in addition to the quantitative changes reflected by reduced topoisomerase II levels, there are qualitative alterations in topoisomerase II function in K/VP.5 cells. This conclusion is further supported by measurement of the stability of VP-16-induced topoisomerase II–DNA covalent complexes which was reduced in K/VP.5 cells (Figure 8) and by the demonstration of a 2-fold increase in ATP requirement for drug-induced K/VP.5 cell topoisomerase II–DNA binding and subsequent catalytic activity (Figures 9 and 10).

The selection technique used to obtain K/VP.5 cells, intermittent then continuous low-concentration (0.5 μM) exposure to VP-16, may relate to the dual phenotypic changes in this cell line, i.e. quantitative and qualitative topoisomerase II alterations. Multifactorial resistance characteristics have been reported using intermittent topoisomerase II inhibitor exposures (Long et al., 1991; Sugawara et al., 1991) or stepwise increases in drug exposure (Ferguson et al., 1988; Matsuo et al., 1990; de Jong et al., 1993) to select for resistant cell lines. However, unlike those studies, the multifactorial resistance reported here for K/VP.5 cells does not include reduced intracellular drug accumulation. More discreet mutational or regulatory changes in topoisomerase II have been observed in cells treated with relatively high concentrations of topoisomerase II inhibitory drugs or with mutagens (Bugg et al., 1991; Hinds et al., 1991; Lee et al., 1992; Chan et al., 1993; Danks 1993). Even though our selection procedure used relatively low concentrations of VP-16 (0.5 μM), the fact that K/VP.5 cells have retained 30-fold resistance to VP-16 in the absence of drug for more than 2 years suggests that a stable mutational alteration has occurred during the acquisition of resistance.

At least two targets for mutation which are not mutually exclusive may be considered as sources for the quantitative and qualitative changes of topoisomerase II observed in the K/VP.5 cell line. First, chronic VP-16 exposure may have selected for an alteration in the topoisomerase II gene itself. A mutation of the primary sequence could produce a less stable mRNA, although the published nucleotide sequence for topoisomerase II (Tsai-Pflugfelder et al., 1988) reveals no known consensus mRNA stability determinants (Cleveland & Yen, 1989). Alternatively, a mutation in the topoisomerase II gene may also result in a RNA that is less efficiently transcribed. The consequence of such a mutation would be a reduction in topoisomerase II mRNA and decreased translation. Previously, we reported a reduction in the stability of topoisomerase II mRNA in K/VP.5 cells that parallels the reduction of topoisomerase II mRNA levels (Ritke &

Yalowich, 1993). In addition, no change in topoisomerase II transcription rate was observed in K/VP.5 compared with K562 cells (Ritke & Yalowich, 1993). Mutations in the coding sequence of the topoisomerase II gene could also result in an alteration in the protein conformation or post-translational modification of this enzyme, thus decreasing the stability of its binding to DNA. Our data demonstrating an attenuation of ATP stimulation of VP-16-induced topoisomerase II–DNA binding in resistant cells correspond to previously published data in VM-26-selected resistant CCRF-CEM cells (Danks *et al.*, 1989) and suggest a mutation in or near ATP-binding domains of topoisomerase II consistent with identified topoisomerase II mutations in several other resistant cell lines (Bugg *et al.*, 1991; Hinds *et al.*, 1991; Lee *et al.*, 1992; Chan *et al.*, 1993; Danks *et al.*, 1993) However, topoisomerase II cDNA sequence analysis has revealed no changes in the region which includes consensus nucleotide-binding domains (nucleotides 1134–1597) comparing K562 and K/VP.5 cells (Ritke *et al.*, submitted). In addition, single-strand conformational polymorphism analysis of topoisomerase II cDNA from K/VP.5 cells has uncovered no evidence of mutations (Ritke *et al.*, submitted). Single-strand conformation polymorphism analysis has been used previously to identify point mutations in topoisomerase II cDNA from drug-resistant cell lines (Danks *et al.*, 1993). These results suggest that, despite the stable biochemical changes in K/VP.5 topoisomerase II which implicate mutations in the gene coding for this enzyme, there may be other genetic changes in resistant cells that affect topoisomerase II function. Sequence analysis of the entire coding sequence of K/VP.5 and K562 topoisomerase II cDNA is under way and will definitively reveal whether a point mutation(s) in K/VP.5 cells is related to qualitative and/or quantitative topoisomerase II alterations documented in the present work.

A second target for genetic alteration of VP-16-selected K/VP.5 cells may be a regulator, effector or co-factor of topoisomerase II. Topoisomerase II has been shown to be phosphorylated in intact cells at serine and threonine residues (Saijo *et al.*, 1990; Kroll & Rowe, 1991; Cardenas *et al.*, 1992) and *in vitro* serves as a substrate for casein kinase II, protein kinase C and $p34^{cdc2}$ kinase (Ackerman *et al.*, 1985; 1988; Sahyoun *et al.*, 1986; Cardenas *et al.*, 1992; Devore *et al.*, 1992; Corbett *et al.*, 1993). In addition, the activity and degree of phosphorylation of topoisomerase II has been found to increase during cell cycle progression from $G_1$ to $G_2$–M phase (Heck *et al.*, 1989; Woessner *et al.*, 1991; Saijo & Enomoto, 1992). These studies suggest a protein kinase as a candidate topoisomerase II co-factor and a mutational target in resistant K/VP.5 cells. Mutation and subsequent altered activity of a protein kinase that phosphorylates topoisomerase II might reduce the stability of this enzyme, resulting in a decreased level of protein in resistant cells. A change in topoisomerase II phosphorylation in resistant cells might also influence ATP binding/hydrolysis and affect catalytic activity, as has recently been demonstrated using *Drosophila* topoisomerase II (Corbett *et al.*, 1993). Altered phosphorylation of topoisomerase II in resistant cells may change the stability of topoisomerase II binding to DNA such that VP-16 stabilisation of the protein–DNA complex is compromised. Recent studies in this laboratory indicate that topoisomerase II phosphorylation is reduced at least 2-fold in K/VP.5 compared with parental K562 cells (Ritke *et al.*, submitted). Therefore, altered topoisomerase II phosphorylation in K/VP.5 cells correlates with decreased VP-16-induced topoisomerase II–DNA binding stability in these resistant cells (Figure 8).

Based on studies presented here, we conclude that selection for a low level of resistance to VP-16 resulted in a quantitative reduction of topoisomerase II expression as well as distinct qualitative changes affecting VP-16-induced stability of topoisomerase II–DNA binding. The multiple stable phenotypic changes exhibited by the novel K/VP.5 cell line provide an opportunity to increase our understanding of the post-transcriptional and/or post-translational modifications in DNA topoisomerase II which regulate its activity in acquired drug resistance.

This work was supported in part by Research Grants CA-51657 and CA-39426 and Cancer Center Support (Core) Grant CA 21765 from the National Cancer Institute, NIH, Bethesda, MD, USA; American Cancer Society Grant DHP-49; and American Lebanese Syrian Associated Charities (ALSAC), M.K.R. is the recipient of a Pittsburgh Cancer Institute Postdoctoral Fellowship.

**Abbreviations**: Topoisomerase II, DNA topoisomerase II; VP-16 (etoposide), 4′-demethylepipodophyllotoxin 9-(4,6-*O*-ethylidene-β-D-glucopyranoside); VM-26 (teniposide), 4′-demethylepipodophyllotoxin 9-(4,6-*O*-2-thenylidene-β-D-glucopyransoide); *m*-AMSA (amsacrine), 4′-(9-acridinylamino)methane-sulphon-*m*-anisidide; SSB, single-strand break; DPC, DNA–protein cross-links; SDS, sodium dodecyl sulphate; DMSO, dimethylsuphoxide; EDTA, ethylene diamine tetraacetic acid; EGTA, ethyleneglycol-bis-(β-aminoethyl ether) *N,N,N′,N′*,-tetraacetic acid.

### References

ACKERMAN, P., GLOVER, C.V.C. & OSHEROFF, N. (1985). Phosphorylation of DNA topoisomerase II by casein kinase II: modulation of eukaryotic topoisomerase II activity in vitro. *Proc. Natl Acad. Sci. USA*, **82**, 3164–3168.

ACKERMAN, P., GLOVER, C.V.C. & OSHEROFF, N. (1988). Phosphorylation of DNA topoisomerase II in vivo and in total homogenates of Drosophila Kc cells. The role of casein kinase II. *J. Biol. Chem.*, **263**, 12653–12660.

BERAN, M. & ANDERSON, B.S. (1987). Development and characterization of a human myelogenous leukemia cell line resistant to 4′-(9-acridinylamino)-3-methane-sulfon-*m*-anisidide. *Cancer Res.*, **47**, 1897–1904.

BUGG, B.Y., DANKS, M.K., BECK, W.T. & SUTTLE, D.P. (1991). Expression of a mutant DNA topoisomerase II in CCRF-CEM human leukemic cells selected for resistance to teniposide. *Proc. Natl Acad. Sci. USA*, **88**, 7654–7658.

CARDENAS, M.E., DANG, Q., GLOVER, C.V.C. & GASSER, S.M. (1992). Casein kinase II phosphorylates the eukaryotic-specific C-terminal domain of topoisomerase II *in vivo*. *EMBO J.*, **11**, 1785–1796.

CHAN, V.T.W., NG, S.-W., EDER, J.P. & SCHNIPPER, L.E. (1993). Molecular cloning and identification of a point mutation in the topoisomerase II cDNA from an etoposide-resistant chinese hamster ovary cell lines. *J. Biol. Chem.*, **268**, 2160–2165.

CHARCOSSET, J.-Y., SAUCIER, J.-M. & JACQUEMIN-SABLON, A. (1991). Reduced DNA topoisomerase II activity and drug-stimulated DNA cleavage in 9-hydroxyellipticine resistant cells. *Biochem. Pharmacol.*, **37**, 2145–2149.

CHEN, G.L., YANG, L., ROWE, T.C., HALLIGAN, B.D., TEWEY, K.M. & LIU, L.F. (1984). Nonintercalative antitumor drugs interfere with the breakage–reunion reaction of mammalian DNA topoisomerase II. *J. Biol. Chem.*, **259**, 13560–13566.

CHOMCZYNSKI, P. & SACCHI, N. (1987). Single-step method of RNA isolation by acid guanidinium thiocyanate–phenol–chloroform extraction. *Anal. Biochem.*, **162**, 156–159.

CLEVELAND, D.W. & YEN, T.J. (1989). Multiple determinants of eukaryotic mRNA stability. *New Biol.*, **1**, 121–126.

COLE, S.P.C., CHANDA, E.R., DICKE, F.P., GERLACH, J.H. & MIRSKI, S.E.L. (1991). Non-P-glycoprotein-mediated multidrug resistance in a small cell lung cancer cell line: evidence for decreased susceptibility to drug-induced DNA damage and reduced levels of topoisomerase II. *Cancer Res.*, **51**, 3345–3352.

CHUNG, T.D.Y., DRAKE, F.H., TAN, K.B., PER, S.R., CROOKE, S.T. & MIRABELLI, C.K. (1989). Characterization and immunological identification of cDNA clones encoding two human DNA topoisomerase II isozymes. *Proc. Natl Acad. Sci. USA*, **86**, 9431–9435.

CORBETT, A.H., FERNALD, A.W. & OSHEROFF, N. (1993). Protein kinase C modulates the catalytic activity of topoisomerase II by enhancing the rate of ATP hydrolysis: evidence for a common mechanism of regulation by phosphorylation. *Biochemistry*, **32**, 2090–2097.

DANKS, M.K., YALOWICH, J.C. & BECK, W.T. (1987). Atypical multiple drug resistance in human leukemic cell lines selected for resistance to teniposide (VM-26). *Cancer Res.*, **47**, 1297–1301.

DANKS, M.K., SCHMIDT, C.A., CIRTAIN, M.C., SUTTLE, D.P. & BECK, W.T. (1988). Altered catalytic activity of and DNA cleavage by DNA topoisomerase II from human leukemic cells selected for resistance to VM-26. *Biochemistry*, **27**, 8861–8869.

DANKS, M.K., SCHMIDT, C.A., DENEKA, D.A. & BECK, W.T. (1989). Increased ATP requirement for activity of and complex formation by DNA topoisomerase II from human leukemic CCRF-CEM cells selected for resistance to teniposide. *Cancer Commun.*, **1**, 101–109.

DANKS, M.K., WARMOTH, M.R., FRICHE, E., GRANZEN, B., BUGG, B.Y., HARKER, W.G., ZWELLING, L.A., FUTSCHER, B.W., SUTTLE, D.P. & BECK, W.T. (1993). Single-strand conformational polymorphism analysis of the $M_r$ 170,000 isozyme of DNA topoisomerase II in human tumor cells. *Cancer Res.*, **53**, 1373–1379.

DAVIES, S.M., ROBSON, C.N., DAVIES, S.L. & HICKSON, I.D. (1988). Nuclear topoisomerase II levels correlate with the sensitivity of mammalian cells to intercalating agents and epipodophyllotoxins. *J. Biol. Chem.*, **263**, 17724–17729.

DEFFIE, A.M., BATRA, J.K. & GOLDENBERG, G.J. (1989). Direct correlation between DNA topoisomerase II activity and cytotoxicity in adriamycin-sensitive and -resistant P388 leukemia cell lines. *Cancer Res.*, **49**, 58–62.

DE JONG, S., LOOISTRA, A.J., DE VRIES, E.G.E., MULDER, N.H. & ZIJLSTRA, J.G. (1993). Topoisomerase II as a target of VM-26 and 4′-(9-acridinylamino)methane-sulfon-*m*-anisidide in atypical multidrug resistant human small cell lung carcinoma cells. *Cancer Res.*, **53**, 1064–1071.

DEVORE, R.F., CORBETT, A.H. & OSHEROFF, N. (1992). Phosphorylation of topoisomerase II by casein kinase II and protein kinase C: effects on enzyme-mediated DNA cleavage/religation and sensitivity to the antineoplastic drug etoposide and 4′-(9-acridinylamino)methane-sulfon-m-anisidide. *Cancer Res.*, **52**, 2156–2161.

DRAKE, F.H., ZIMMERMAN, J.P., MCCABE, F.L., BASTUS, H.F., PER, S.R., SULLIVAN, D.M., ROSS, W.E., MATTERN, M.R., JOHNSON, R.K., CROOKE, S.T. & MIRABELLI, C.K. (1987). Purification of topoisomerase II from amsacrine-resistant P388 leukemia cells. *J. Biol. Chem.*, **262**, 16739–16747.

DRAKE, F.H., HOFMANN, G.A., BARTUS, H.F., MATTERN, M.R., CROOKE, S.T. & MIRABELLI, C.K. (1989). Biochemical and pharmacological properties of p170 and p180 forms of topoisomerase II. *Biochemistry*, **28**, 8154–8160.

FEINBERG, A.P. & VOGELSTEIN, B. (1983). A technique for radiolabeling DNA restriction fragments to high specific activity. *Anal. Biochem.*, **137**, 266–267.

FERGUSON, P.J., FISHER, M.H., STEPHENSON, J., LI, D.-H., ZHOU, B.-S. & CHENG, Y.-C. (1988). Combined modalities of resistance in etoposide-resistant human KB cell lines. *Cancer Res.*, **48**, 5956–5964.

FERNANDES, D.J., DANKS, M.K. & BECK, W.T. (1990). Decreased nuclear matrix DNA topoisomerase II in human leukemia cells resistant to VM-26 and m-AMSA. *Biochemistry*, **29**, 4235–4241.

FILIPSKI, J. & KOHN, K.W. (1982). Ellipticine-induced protein-associated DNA breaks in isolated L1210 nuclei. *Biochim. Biophys. Acta*, **698**, 280–286.

FRICHE, E., DANKS, M.K., SCHMIDT, C.A. & BECK, W.T. (1991). Decreased DNA topoisomerase II in daunorubicin-resistant Ehrlich ascites tumor cells. *Cancer Res.*, **51**, 4213–4218.

GLISSON, B., SMALLWOOD, S. & ROSS, W.E. (1984). Characterization of VP-16-induced DNA damage in isolated nuclei from L1210 cells. *Biochim. Biophys. Acta*, **783**, 74–79.

GLISSON, B., GUPTA, R., SMALLWOOD-KENTRO, S. & ROSS, W. (1986). Characterization of acquired epipodophyllotoxin resistance in a Chinese hamster ovary cell line: loss of drug-stimulated DNA cleavage activity. *Cancer Res.*, **46**, 1934–1938.

GUPTA, R.S. (1983). Genetic, biochemical, and cross-resistance studies with mutants of Chinese hamster ovary cells resistant to the anticancer drugs, VM-26 and VP-16-213. *Cancer Res.*, **43**, 1568–1574.

HARKER, W.G., SLADE, D.L., DALTON, W.S., MELTZER, P.S. & TRENT, J.M. (1989). Multidrug resistance in mitoxantrone-selected HL-60 leukemia cells in the absence of P-glycoprotein overexpression. *Cancer Res.*, **49**, 4542–4549.

HARKER, W.G., SLADE, D.L., DRAKE, F.H. & PARR, R.L. (1991). Mitoxantrone resistance in HL-60 leukemia cells: reduced nuclear topoisomerase II catalytic activity and drug-induced DNA cleavage in association with reduced expression of the topoisomerase II $\beta$ isoform. *Biochemistry*, **30**, 9953–9961.

HECK, M.M.S., HITTELMAN, W.N. & EARNSHAW, W.C. (1989). In vivo phosphorylation of the 170-kDa form of eukaryotic DNA topoisomerase II: cell cycle analysis. *J. Biol. Chem.*, **264**, 15161–15164.

HINDS, M., DEISSEROTH, K., MAYES, J., ALTSCHULER, E., JANSEN, R., LEDLEY, F.D. & ZWELLING, L.A. (1991). Identification of a point mutation in the topoisomerase II gene from a human leukemia cell line containing an Amsacrine-resistant form of topoisomerase II. *Cancer Res.*, **51**, 4729–4731.

KAMATH, N., GRABOWSKI, D., FORD, J., KERRIGAN, D., POMMIER, Y. & GANAPATHI, R. (1992). Overexpression of P-glycoprotein and alterations in topoisomerase II in P388 mouse leukemia cells selected in vivo for resistance to mitoxantrone. *Biochem. Pharmacol.*, **44**, 937–945.

KOHN, K.W., ERICKSON, L.C., EWIG, R.A.G. & FRIEDMAN, C.A. (1976). Fractionation of DNA from mammalian cells by alkaline elution. *Biochemistry*, **14**, 4629–4637.

KROLL, D.J. & ROWE, T.C. (1991). Phosphorylation of DNA topoisomerase II in a human tumor cell line. *J. Biol. Chem.*, **266**, 7957–7961.

LAEMMLI, U.K. (1970). Cleavage of structural proteins during the assembly of the head of bacteriophage. *Nature*, **227**, 680–685.

LEE, M.-S., WANG, J.C. & BERAN, M. (1992). Two independent amsacrine-resistant human myeloid leukemia cell lines share an identical point mutation in the 170 kDa form of human topoisomerase II. *J. Mol. Biol.*, **223**, 837–843.

LIU, L.F. (1989). DNA topoisomerase poisons as antitumor drugs. *Annu. Rev. Biochem.*, **58**, 351–375.

LONG, B.H., WANG, L., LORICO, A., WANG, R.C.C., BRATTAIN, M.G. & CASAZZA, A.M. (1991). Mechanisms of resistance to etoposide and teniposide in acquired resistant human colon and lung carcinoma cell lines. *Cancer Res.*, **51**, 5275–5284.

MANIATIS, T., FRITSCH, E.F. & SAMBROOK, J. (1982). In *Molecular Cloning: A Laboratory Manual.* Cold Spring Harbor Laboratory Press: Cold Spring Harbor, NY.

MATSUO, K., KOHNO, K., TAKANO, H., SATO, S., KIUE, A. & KUWANO, M. (1990). Reduction of drug accumulation and DNA topoisomerase II activity in acquired teniposide-resistant human cancer KB cell lines. *Cancer Res.*, **50**, 5819–5824.

MINFORD, J., POMMIER, Y., FILIPSKI, J., KOHN, K.W., KERRIGAN, D., MATTERN, M., MICHAELS, S., SCHWARTZ, R. & ZWELLING, L.A. (1986). Isolation of intercalator-dependent protein-linked DNA strand cleavage activity from cell nuclei and identification as topoisomerase II. *Biochemistry*, **25**, 9–16.

MIRSKI, S.E.L., EVANS, C.D., ALMQUIST, K.C., SLOVAK, M.L. & COLE, S.P.C. (1993). Altered topoisomerase II$\alpha$ in a drug-resistant small cell lung cancer cell line selected in VP-16. *Cancer Res.*, **53**, 4866–4873.

NELSON, E.M., TEWEY, K.M. & LIU, L.F. (1984). Mechanism of antitumor drug action: poisoning of mammalian DNA topoisomerase II on DNA by 4′(9-acridinylamino)methane-sulfone-m-anisidide. *Proc. Natl Acad. Sci. USA*, **81**, 1361–1365.

NORMAN, M.R. & THOMPSON, E.B. (1977). Characterization of a glucocorticoid-sensitive human lymphoid cell line. *Cancer Res.*, **37**, 3785–3791.

ODAIMI, M., ANDERSON, B.S., MCCREDIE, K.B. & BERAN, M. (1986). Drug sensitivity and cross-resistance of the 4′-(9-acridinylamino)methane sulfon-m-anisidide-resistant subline of HL-60 human leukemia. *Cancer Res.*, **46**, 3330–3333.

OSHEROFF, N. (1989). Biochemical basis for the interactions of type I and type II topoisomerases with DNA. *Pharmacol. Ther.*, **41**, 223–241.

PATEL, S. & FISHER, L.M. (1993). Novel selection and genetic characterisation of an etoposide-resistant human leukaemic CCRF-CEM cell line. *Br. J. Cancer*, **67**, 456–463.

PER, S.R., MATTERN, M.R., MIRABELLI, C.K., DRAKE, F.H., JOHNSON, R.K. & CROOKE, S.T. (1987). Characterization of a subline of P388 leukemia resistant to amsacrine: evidence of altered topoisomerase II function. *Mol. Pharmacol.*, **32**, 17–25.

POLITI, P.M., ARNOLD, S.T., FELSTED, R.L. & SINHA, B.K. (1990). P-glycoprotein-independent mechanism of resistance to VP-16 in multidrug-resistant tumor cell lines: pharmacokinetic and photo-affinity labeling studies. *Mol. Pharmacol.*, **37**, 790–796.

POMMIER, Y., SCHWARTZ, R.E., KOHN, K.W. & ZWELLING, L.A. (1984). Formation and rejoining of deoxyribonucleic acid double-strand breaks induced in isolated cell nuclei by antineoplastic intercalating agents. *Biochemistry*, **23**, 3194–3201.

POMMIER, Y., SCHWARTZ, R.E., ZWELLING, L.A., KERRIGAN, D., MATTERN, M.R., CHARCOSSET, J.-Y., JACQUENIN-SABLON, A. & KOHN, K.W. (1986*a*). Reduced formation of protein-associated DNA strand breaks in Chinese hamster cells resistant to topoisomerase inhibitors. *Cancer Res.*, **46**, 611–616.

POMMIER, Y., KERRIGAN, D., SCHWARTZ, R.E., SWACK, J.A. & MCCURDY, A. (1986*b*). Altered DNA topoisomerase II activity in Chinese hamster cells resistant to topoisomerase II inhibitors. *Cancer Res.*, **46**, 3075–3081.

RITKE, M.K. & YALOWICH, J.C. (1993). Altered gene expression in human leukemia K562 cells selected for resistance to etoposide (VP-16). *Biochem. Pharmacol.*, **46**, 2007–2020.

RITKE, M.K., ALLAN, W.P., GUNDUZ, N.N. & YALOWICH, J.C. (1993). Reduced phosphorylation of topoisomerase II in etoposide-resistant human leukemia K562 cells. *Mol. Pharmacol.* (submitted).

ROBERTS, D., FOGLESONG, P.D., PARGANAS, E. & WIGGINS, L. (1989). Reduced formation of lesions in the DNA of a multidrug-resistant L1210 subline selected for teniposide resistance. *Cancer Chemother. Pharmacol.*, **23**, 161–168.

ROSS, W.E., GLAUBIGER, D. & KOHN, K.W. (1979). Qualitative and quantitative aspects of intercalator-induced DNA strand breaks. *Biochim. Biophys. Acta*, **562**, 41–50.

SAHAI, B.M. & KAPLAN, J.G. (1986). A quantitive decatenation assay for type II topoisomerases. *Anal. Biochem.*, **156**, 364–379.

SAHYOUN, N., WOLF, M., BESTERMAN, J., HSIEH, T.-S., SANDER, M., LEVINE, H., CHANG, K.-J. & CUATRECASAS, P. (1986). Protein kinase C phosphorylates topoisomerase II: topoisomerase activation and its possible role in phorbol ester-induced differentiation of HL-60 cells. *Proc. Natl Acad. Sci. USA*, **83**, 1603–1607.

SAIJO, M., UI, M. & ENOMOTO, T. (1992). Growth state and cell cycle dependent phosphorylation of DNA topoisomerase II in Swiss 3T3 cells. *Biochemistry*, **31**, 359–363.

SAIJO, M., ENOMOTO, T., HANAOKA, F. & UI, M. (1990). Purification and characterization of type II DNA topoisomerase from mouse FM3A cells: phosphorylation of topoisomerase II and modification of its activity. *Biochemistry*, **29**, 583–590.

SINKULE, J.A. & EVANS, W.E. (1984). High performance liquid chromatographic analysis of the semisynthetic epipodophyllotoxins teniposide and etoposide using electrochemical detection. *J. Pharmaceut. Sci.*, **73**, 164–168.

SUGAWARA, I., IWAHASHI, T., OKAMOTO, K., SUGIMOTO, Y., EKIMOTO, H., TSURUO, T., IKEUCHI, T. & MORI, S. (1991). Characterization of an etoposide-resistant human K562 cell line, K/eto. *Jpn. J. Cancer Res.*, **82**, 1035–1043.

SULLIVAN, D.M., LATHAM, M.D., ROWE, T.C. & ROSS, W.E. (1989). Purification and characterization of an altered topoisomerase II from a drug-resistant Chinese hamster ovary cell line. *Biochemistry*, **28**, 5680–5687.

SULLIVAN, D.M., ESKILDSEN, L., GROOM, K.R., WEBB, C.D., LATHAM, M.D., MARTIN, A.W., WELLHAUSEN, S.R., KROEGER, P.E. & ROWE, T.C. (1993). Topoisomerase II activity involved in cleaving DNA into topological domains is altered in a multiple drug-resistant chinese hamster ovary cell line. *Mol. Pharmacol.*, **43**, 207–216.

TAKANO, H., KOHNO, K., ONO, M., UCHIDA, Y. & KUWANO, M. (1991). Increased phosphorylation of DNA topoisomerase II in etoposide-resistant mutants of human cancer KB cells. *Cancer Res.*, **51**, 3951–3957.

TEWEY, K.M., ROWE, T.C., YANG, L., HALLIGAN, B.D. & LIU, L.F. (1984*a*). Adriamycin-induced DNA damage mediated by mammalian DNA topoisomerase II. *Science*, **226**, 466–468.

TEWEY, K.M., CHEN, G.L., NELSON, E.M. & LIU, L.F. (1984*b*). Intercalating antitumor drugs interfere with the breakage-reunion reaction of mammalian DNA topoisomerase II. *J. Biol. Chem.*, **259**, 9182–9187.

TOWBIN, H., STAEHELIN, T. & GORDON, J. (1979). Electrophoretic transfer of proteins from polyacrylamide gels to nitrocellulose sheets: procedures and some applications. *Proc. Natl Acad. Sci. USA*, **76**, 4350–4354.

TSAI-PFLUGFELDER, M., LIU, L.F., LIU, A.A., TEWEY, K.M., WHANG-PENG, J., KNUTSEN, T., HUEBER, K., CROCE, C.M. & WANG, J.C. (1988). Cloning and sequencing of cDNA encoding human DNA topoisomerase II and localization of the gene to chromosome region 17q21-22. *Proc. Natl Acad. Sci. USA*, **85**, 7177–7181.

WANG, J.C. (1985). DNA topoisomerases. *Annu. Rev. Biochem.*, **54**, 665–697.

WEBB, C.D., LATHAN, M.D., LOCK,, R.B. & SULLIVAN, D.M. (1991). Attenuated topoisomerase II content directly correlates with a low level of drug resistance in a chinese hamster ovary cell line. *Cancer Res.*, **51**, 6542–6549.

WEBB, C.F., ENEFF, K.L. & DRAKE, F.H. (1993). A topoisomerase II-like protein is part of an inducible DNA-binding protein complex that binds 5′ of an immunoglobulin promoter. *Nucleic Acids Res.*, **21**, 4363–4368.

WOESSNER, R.D., MATTERN, M.R., MIRABELLI, C.K., JOHNSON, R.K. & DRAKE, F.H. (1991). Proliferation- and cell cycle-dependent differences in expression of the 170 kilodalton and 180 kilodalton forms of topoisomerase II in NIH-3T3 cells. *Cell Growth Different.*, **2**, 209–214.

YALOWICH, J.C. (1987). Effects of microtubule inhibitors on etoposide accumulation and DNA damage in human K562 cells *in vitro*. *Cancer Res.*, **47**, 1010–1015.

YALOWICH, J.C. & GOLDMAN, I.D. (1984). Analysis of the inhibitory effects of VP-16-213 (etoposide) and podophyllotoxin on thymidine transport and metabolism in Ehrlich ascites tumor cells *in vitro*. *Cancer Res.*, **44**, 984–989.

ZHANG, H., D'ARPA, P. & LIU, L.F. (1990). A model for tumor cell killing by topoisomerase poisons. *Cancer Cells*, **2**, 23–27.

ZWELLING, L.A. (1985). DNA topoisomerase II as a target of antineoplastic drug therapy. *Cancer Metastasis Rev.*, **4**, 263–276.

ZWELLING, L.A., HINDS, M., CHAN, D., MAYES, L., LAN SIE, K., PARKER, E., SILBERMAN, L., RADCLIFFE, A., BERAN, M. & BLICK, M. (1989). Characterization of an amsacrine-resistant line of human leukemia cells: evidence for a drug-resistant form of topoisomerase II. *J. Biol. Chem.*, **264**, 16411–16420.